# Design and Preclinical Evaluation of Novel uPAR-Targeting Radiopeptides Modified with an Albumin-Binding Entity

**DOI:** 10.1021/acs.molpharmaceut.5c00135

**Published:** 2025-05-06

**Authors:** Darja Beyer, Christian Vaccarin, Jerome V. Schmid, Luisa M. Deberle, Xavier Deupi, Roger Schibli, Cristina Müller

**Affiliations:** † Center for Radiopharmaceutical Sciences, 28498 PSI Center for Life Sciences, Forschungsstrasse 111, 5232 Villigen-PSI, Switzerland; ‡ Condensed Matter Theory Group, PSI Center for Scientific Computing, Theory, and Data, 5232 Villigen-PSI, Switzerland; § Laboratory of Biomolecular Research, 28498 PSI Center for Life Sciences, 5232 Villigen-PSI, Switzerland; ∥ Swiss Institute of Bioinformatics (SIB), 1015 Lausanne, Switzerland; ⊥ Department of Chemistry and Applied Biosciences, ETH Zurich, 8093 Zurich, Switzerland

**Keywords:** urokinase-type plasminogen activator receptor, uPAR targeting, SPECT imaging, lutetium-177, albumin binder, iodophenyl entity, AE105

## Abstract

Several studies have focused on the development and application of radiolabeled DOTA-AE105 for targeting the urokinase-type plasminogen activator receptor (uPAR), which is expressed on various cancer types. The aim of this project was to design and evaluate novel uPAR-targeting radiopeptides with improved pharmacokinetic properties in view of their therapeutic application. Five peptides (uPAR-01, uPAR-02, uPAR-03, uPAR-04, and uPAR-05) were synthesized based on the AE105 peptide backbone, a DOTA chelator, and the 4-(*p*-iodophenyl)­butanoate moiety as an albumin binder. The peptides were obtained in 20–29 synthetic steps using solid-phase peptide synthesis with a 6–34% overall yield. In saline, the ^177^Lu-labeled peptides (100 MBq/nmol) were stable (>93% intact radiopeptides) in the presence of l-ascorbic acid over 24 h. The new radiopeptides were also stable (>98% intact radiopeptides) in mouse and human blood plasma, while only ∼13% of [^177^Lu]­Lu-DOTA-AE105 was intact after a 4 h incubation period. The uPAR-binding affinities (*K*_D_ values) determined with uPAR-transfected human embryonic kidney cells (HEK-uPAR) ranged from 10 to 57 nM and were, thus, similar to that of [^177^Lu]­Lu-DOTA-AE105 (*K*_D_: 20 ± 1 nM). Compared to [^177^Lu]­Lu-DOTA-AE105, the radiopeptides showed the anticipated increased binding affinity to plasma proteins both in mouse (31- to 104-fold) and human blood plasma (43- to 136-fold). The tissue distribution of the novel radiopeptides in nude mice bearing HEK-uPAR xenografts showed substantial activity retention in the blood (12–16% IA/g and 4.5–13% IA/g at 4 and 24 h p.i., respectively), while [^177^Lu]­Lu-DOTA-AE105 was rapidly cleared (<0.1% IA/g at 4 h p.i.). As a result, the accumulation of the new radiopeptides in HEK-uPAR xenografts (3.6–11% and 3.1–10% IA/g at 4 and 24 h p.i., respectively) was increased in comparison to that of [^177^Lu]­Lu-DOTA-AE105 (<1% IA/g at 4 h p.i.). Importantly, the metabolic stability of the new radiopeptides in mice was enhanced as compared to that of [^177^Lu]­Lu-DOTA-AE105. [^177^Lu]­Lu-uPAR-02 showed the most promising tissue distribution profile with over 10-fold higher activity retention in the HEK-uPAR xenograft than observed after injection of [^177^Lu]­Lu-DOTA-AE105. As a result, the xenograft-to-kidney ratio of [^177^Lu]­Lu-uPAR-02 was >3-fold higher than that of [^177^Lu]­Lu-DOTA-AE105.

## Graphical abstract

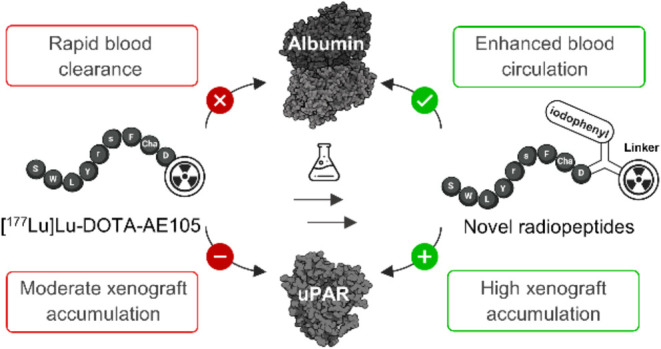

## Introduction

The human urokinase-type plasminogen activator receptor (uPAR) is a glycosylphosphatidylinositol membrane-anchored protein,^1^ expressed in numerous solid tumors, including breast, gastric, pancreatic, colorectal, prostate, ovarian, oral and esophageal cancer.^2–5^ Its presence has been associated with tumorigenesis, metastasis, angiogenesis, tumor proliferation, and invasion.^6,7^ Importantly, uPAR was also found expressed on extracellular matrix components, including angiogenic endothelial cells and macrophages.^8^ In normal tissue, this receptor is not commonly expressed; however, it is transiently present during wound healing.^8^

The expression pattern of uPAR makes it a relevant tumor-associated marker for targeting with diagnostic and therapeutic radiopharmaceuticals. Previously, a synthetic uPAR-binding nonapeptide, referred to as AE105, was identified using phage display methodologies and affinity maturation techniques.^9,10^ The reported strong binding affinity to human uPAR (*K*_D_ = 0.4 nM) made AE105 an interesting candidate for the development of nuclear imaging agents and radiotherapeutics.^9,11^ AE105 was conjugated with various macrocyclic chelators to enable the coordination of copper-64, gallium-68, and lutetium-177.^12^ [^64^Cu]­Cu-DOTA-AE105 was successfully used to visualize small foci in a mouse model of disseminated prostate cancer,^13^ while [^68^Ga]­Ga-DOTA-AE105 and [^68^Ga]­Ga-NODAGA-AE105 were investigated in PET imaging studies of U78MG tumor-bearing mice.^14^

In several clinical trials (e.g., NCT02139371 and NCT06474806), [^64^Cu]­Cu-DOTA-AE105 was tested for PET imaging of breast, urinary bladder, and prostate cancer.^15^ [^68^Ga]­Ga-NOTA-AE105 was employed for PET imaging of patients with neuroendocrine tumors (NCT03278275), breast cancer (NCT02681640), castration-resistant prostate cancer (NCT02964988), and head and neck cancer (NCT02965001).^16,17^ The suitability of AE105-based radiopeptides for therapeutic applications has not yet been investigated in clinics. Preclinically, [^177^Lu]­Lu-DOTA-AE105 showed limited efficacy in delaying tumor growth in mice, presumably due to its rapid renal excretion and, as a result, low tumor accumulation.^18,19^

The concept of modifying radiopharmaceuticals with an albumin-binding entity to enhance their blood circulation time and, therewith, the tumor uptake^20^ has been exemplified with a variety of tumor-targeting agents,^20^ including folate radioconjugates,^21–23^ prostate-specific membrane antigen (PSMA)-targeting radioligands,^24–30^ and somatostatin analogues.^31^ These preclinical studies indicate the necessity of identifying the optimal albumin binder and spacer entity separately for each tumor-targeting agent through systematic investigations to achieve the desired distribution profile.

The aim of this study was to develop novel AE105-based radiopeptides modified with an albumin-binding entity to enhance their pharmacokinetic properties for potential use in radionuclide therapy (Figure 1). The introduction of a lysine residue between the AE105 peptide backbone and the DOTA chelator enabled the conjugation of the *p*-iodophenyl entity via the lysine’s Nε group to obtain uPAR-01. The implementation of a hydrophilic PEG_4_ spacer next to the *p*-iodophenyl entity in uPAR-02 was expected to moderately reduce the albumin-binding affinity as previously shown with folate radioconjugates^22^ and PSMA radioligands.^26^ In contrast, the introduction of the lipophilic aminomethyl benzoic acid (AMBA) entity next to the albumin binder, as exemplified in uPAR-03, was thought to increase the albumin-binding affinity as it was previously demonstrated with folate radioconjugates.^23,32^ In uPAR-04 and uPAR-05, a lysine residue or PEG_4_ spacer, respectively, was introduced between the AE105 peptide backbone and the DOTA chelator with the intention to increase their uPAR-binding affinity.

**Figure 1:**
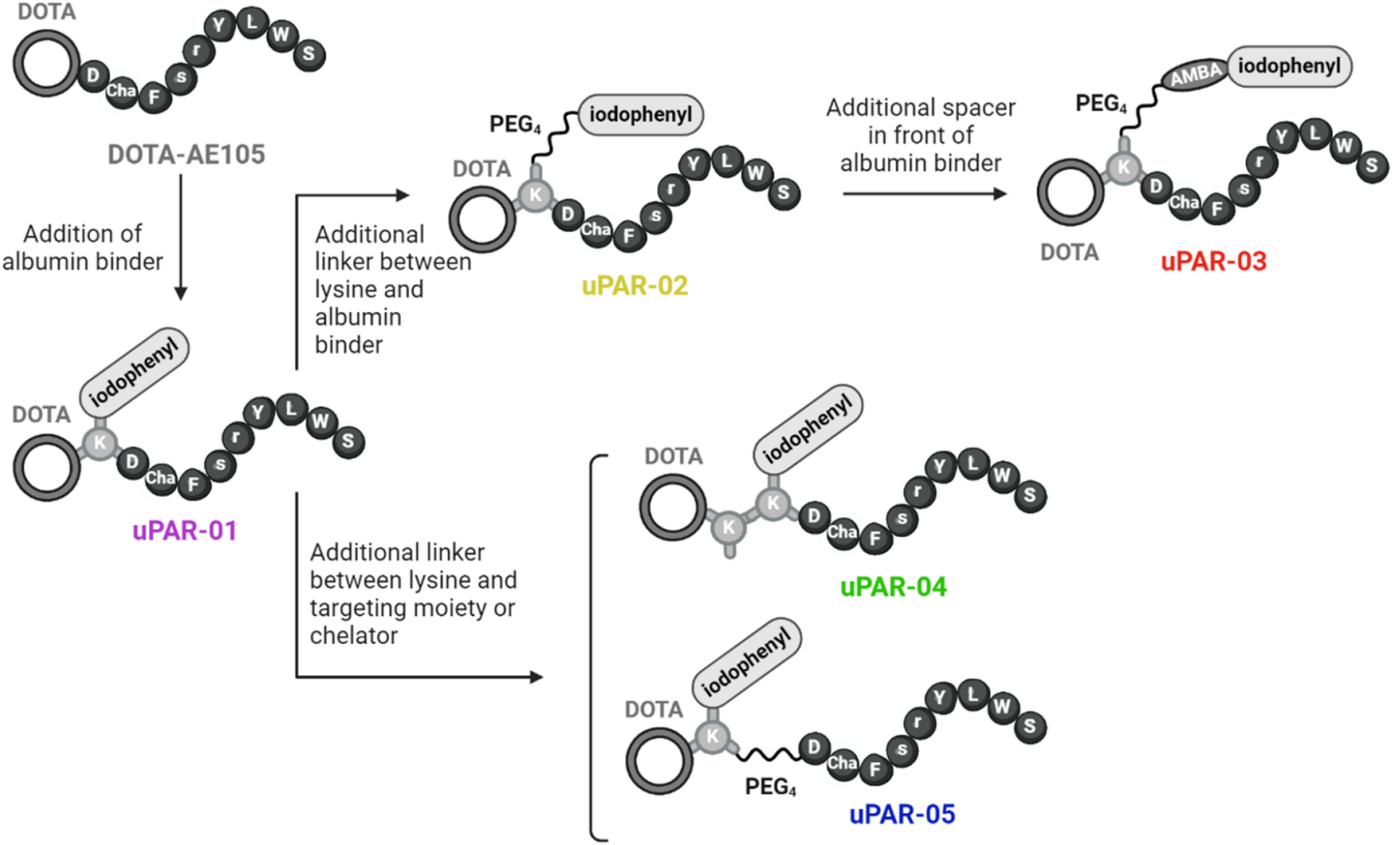
Overview of the design of AE105-based uPAR-targeting radiopeptides using variable linker entities and interconnectivities.

Considering their future therapeutic application, these new uPAR-targeting peptides were radiolabeled with lutetium-177. The resultant radiopeptides, [^177^Lu]­Lu-uPAR-01, [^177^Lu]­Lu-uPAR-02, [^177^Lu]­Lu-uPAR-03, [^177^Lu]­Lu-uPAR-04, and [^177^Lu]­Lu-uPAR-05, were investigated in the preclinical setting using uPAR-transfected HEK cells and xenograft-bearing mice.

## Material and Methods

### Synthesis of uPAR-Targeting Peptides

The nona­peptide AE105 and its derivative DOTA-AE105 were synthesized via Fmoc solid-phase peptide synthesis according to a previously reported procedure, with minor modifications (Scheme S1).^15^ A synthesis intermediate, represented by the fully protected and resin-immobilized AE105 (RI-AE105) scaffold, was used as the common starting material for the production of the newly designed peptides equipped with an albumin-binding entity (Scheme 1 and Figure S1). uPAR-01 was prepared by conjugation of a lysine residue to the *N*α-terminus of RI-AE105 in order to obtain a bifurcation point. The *N*ε-amino group of the terminal lysine was then conjugated with 4-(*p*-iodophenyl)­butanoic acid, while, after removal of the Dde protecting group, the Nα-amine was coupled to 2-[4,7,10-tris­[2-[(2-methylpropan-2-yl)­oxy]-2-oxoethyl]-1,4,7,10-tetrazacyclododec-1-yl]­acetic acid (DOTA-tris­(^t^Bu)­ester) (Scheme S2). All other uPAR-targeting peptides were prepared following a reaction sequence similar to the one used for the synthesis of uPAR-01; however, additional spacers were introduced next to the albumin binder or the AE105 peptide scaffold. uPAR-02 and uPAR-03 were prepared by introducing 3-[2-[2-[2-(2-aminoethoxy)­ethoxy]­ethoxy]­ethoxy]­propanoic acid (PEG_4_) alone or in addition to a 4-(aminomethyl)­benzoic acid (AMBA) entity, respectively, before the insertion of the albumin-binding group (Schemes S3 and S4). In the case of uPAR-04, an additional lysine residue was introduced before the conjugation of the DOTA macrocyclic chelator to the Nε-amino group (Scheme S5). uPAR-05 was prepared by introducing a PEG_4_ spacer between the AE105 backbone and the lysine residue that served as the bifurcation point to connect the albumin binder and the macrocyclic DOTA chelator with the AE105 scaffold (Scheme S6). The respective molecules were treated with a solution of trifluoroacetic acid (TFA) containing 2.5% (v/v) water and 2.5% (v/v) triisopropylsilane as radical scavengers to cleave them from the resin and simultaneously removed the acid-labile protecting groups. The crude products were purified using semipreparative high-performance liquid chromatography (HPLC) to isolate the desired peptides (Table S1). The chemical purity of the final products was determined using analytical HPLC equipped with a UV-detector (λ = 254 nm). The chemical identity of the AE105-based peptides was confirmed by high-resolution mass spectrometry (HRMS) analysis (Supporting Information).

**Scheme 1:**
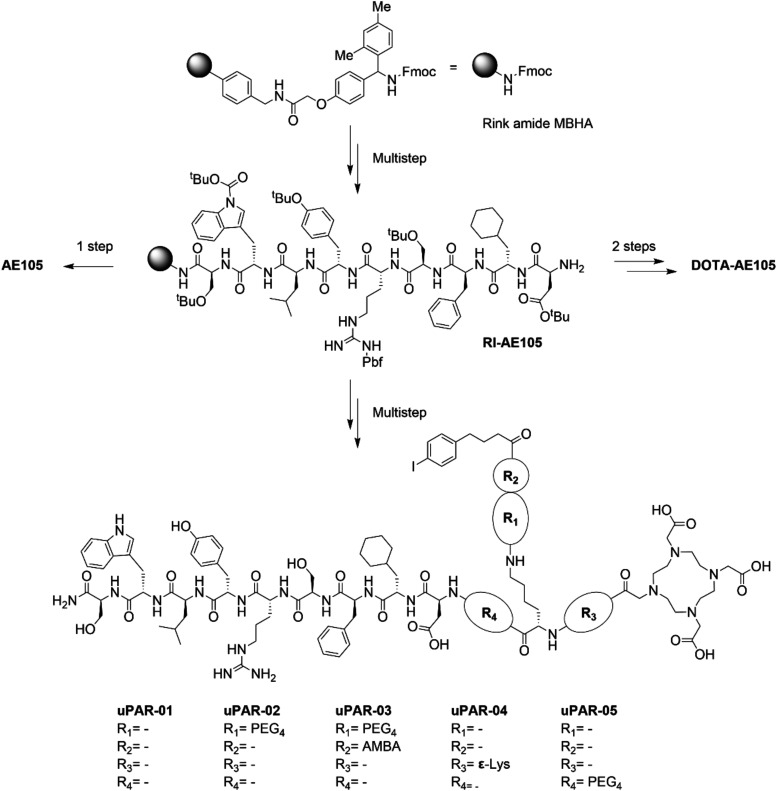
Synthesis Scheme of the uPAR-Targeting Peptides

### Radiolabeling of the uPAR-Targeting Peptides and In Vitro Stability

The radiolabeling of the uPAR-targeting peptides was performed using no-carrier-added lutetium-177 (EndolucinBeta, n.c.a. [^177^Lu]­LuCl_3_ in 0.04 M HCl; ITM Medical Isotopes GmbH, Germany). The respective amount of peptide (stock solution of 1 mM) and lutetium-177 were added to a 1/5 (v/v) mixture of sodium acetate (0.5 M) and HCl (0.05 M), followed by incubation of the reaction mixture at 95 °C for 10 min (Supporting Information). The quality control of the prepared radiopeptides was performed using HPLC.

The radiolytic stability of the radiopeptides (50 MBq/nmol) in saline (150 MBq/300 μL) was assessed in the absence and presence of l-ascorbic acid (3 mg added in a volume of 20 μL). After an incubation period of 1, 4, and 24 h, aliquots of the solutions were analyzed using HPLC. The integrated peak area representing the intact radiopeptide was expressed as the percentage of the sum of all peaks of the chromatogram. The final amount of intact radiopeptide was expressed as the percentage of the product peak obtained at *t* = 0, which was set as 100% (Supporting Information).

### Stability of the Radiopeptides in Mouse and Human Blood Plasma

The radiopeptides (50 MBq/nmol) stabilized with l-ascorbic acid were diluted in mouse and human blood plasma and incubated at 37 °C. Aliquots were taken after 1, 4, and 24 h and analyzed using reversed-phase thin-layer chromatography (TLC) (Supporting Information).

### *n*-Octanol/PBS Distribution Coefficients (logD Values)

The radiopeptides diluted in phosphate-buffered saline (PBS) pH 7.4 (25 μL, ∼0.5 MBq, 0.01 nmol) were added to a mixture of PBS pH 7.4 (1475 μL) and *n*-octanol (1500 μL). The vials were vortexed vigorously for 1 min, followed by centrifugation (560 rcf, 6 min) for phase separation. Aliquots were taken from each phase and measured in a γ-counter (1480 Wizard, PerkinElmer). The distribution coefficients were calculated as the logarithmic value of the ratio of counts per minute (cpm) measured in the *n*-octanol phase relative to the cpm measured in the PBS phase. The results were listed as average ± standard deviation (SD) of the data obtained from 3 independent experiments, each performed with five replicates.

### Albumin-Binding Properties

The relative albumin-binding affinities of the radiopeptides in mouse and human blood plasma were determined using an ultrafiltration assay with Amicon centrifugal filters (cutoff of 10 kDa; Merck Millipore), as previously reported (Supporting Information).^23^ A fixed amount of the respective radiopeptide (15 μL, ∼300 kBq, 0.006 nmol) was added to a defined volume (150 μL) of mouse and human blood plasma dilutions to obtain [mouse serum albumin (MSA)]-to-[radiopeptide] and [human serum albumin (HSA)]-to-[radiopeptide] molar concentration ratios of 0.01–12,500 and 0.01–20,000, respectively. The data were analyzed using a semilogarithmic Hill plot with the maximum binding set as 100% (GraphPad Prism software, version 8.3.1). The half-maximum binding (*B*_50_ value) of each radiopeptide was expressed as the relative value using the inverse ratios, with the value of [^177^Lu]­Lu-uPAR-01 set as 1.0.

### Cell Culture

HEK cells transfected with human uPAR, herein referred to as HEK-uPAR cells, were obtained from Innoprot, Innovative Technologies in Biological Systems S.L. (Bizkaia, Spain). The cells were cultured in Dulbecco’s modified Eagle’s medium (DMEM) supplemented with 1% nonessential amino acids, 10% fetal calf serum, l-glutamine, and antibiotics. Hygromycin B (50 μg/mL) was added to maintain uPAR expression. The cells were subcultured twice a week using trypsin/ethylenediaminetetraacetic acid and maintained under standard cell culture conditions using 5% CO_2_ at 37 °C in a humidified atmosphere.

### Cell Uptake and Internalization

HEK-uPAR cells were seeded in polylysine-coated 12-well plates. The following day, the cells were incubated with the respective radiopeptide (38 kBq, 0.75 pmol per well) in the absence or presence of excess AE105 (final concentration of 5 μM) to block uPAR. After incubation of the HEK-uPAR cells for 2 or 4 h at 37 °C, they were rinsed with ice-cold PBS only to determine the total cell uptake or additionally with acidic stripping buffer (pH 2.8, 50 mM glycine, 100 mM NaCl) to determine the internalized fraction of the radiopeptides. The cells were lysed with an aqueous NaOH solution (1 M, 1 mL), and the lysates were transferred to radioimmunoassay tubes for counting in the γ-counter (1480 Wizard, PerkinElmer). The values were standardized to the protein amount in each well using a Micro BCA Protein Assay kit (Pierce, Thermo Scientific). The uptake and internalized fraction were expressed as the percentage of total added activity per well and presented as the average ± SD of *n* = 3 independently performed experiments.

### uPAR-Binding Affinity (*K*_D_ Values)

To determine the uPAR-binding affinity of the radiopeptides, saturation experiments were performed using HEK-uPAR cells in 48-well plates (Supporting Information). The cells were incubated on ice for 1 h in the presence or absence of excess AE105 (final concentration of 40 μM) together with the respective radiopeptide at a concentration range of 1–1600 nM. After rinsing the cells with PBS, they were lysed with an aqueous NaOH solution (1 M, 600 μL), transferred to radioimmunoassay tubes, and measured in a γ-counter (1480 Wizard, PerkinElmer). The *K*_D_ values were determined by plotting the specific binding (values of samples incubated without AE105 minus the values of samples incubated with AE105) against the molar concentration of the added peptide. A nonlinear regression analysis was performed using GraphPad Prism software (version 8.3.1). The results were expressed as average ± SD of *n* = 3 independently performed experiments.

### Animal Studies

All applicable international, national, and/or institutional guidelines for the care and use of laboratory animals were followed, and the herein reported experiments were carried out according to the guidelines of the Swiss Regulations for Animal Welfare. The preclinical studies were ethically approved by the Cantonal Committee of Animal Experimentation and permitted by the responsible cantonal authorities (License No. 75721). Five-week-old female CD1/nude (Crl:CD1-*Foxn* ^*nu*^) and female immunocompetent FVB (FVB/NCrl) mice were obtained from Charles River Laboratories (Sulzfeld, Germany). All mice were acclimatized for at least 7 days, receiving standard rodent chow ad libitum before being included in the reported studies. The CD1/nude mice were subcutaneously inoculated with HEK-uPAR cells (7 × 10^6^ cells in 100 μL PBS) on the right shoulder. The growth of the HEK-uPAR xenografts varied significantly between mice. Typically, it took 3–11 weeks to reach xenograft volumes of 40–600 mm^3^ at the time of testing the tissue distribution of the radiopeptides. The immunocompetent FVB mice were used for testing the in vivo stability of the radiopeptides.

### SPECT/CT Imaging Studies

The mice were scanned at 1, 4, and 24 h after injection of the respective radiopeptide (25 MBq, 0.5 nmol, 100 μL), which was diluted in saline containing 0.05% bovine serum albumin (BSA). Imaging studies were performed using a four-head, multiplexing, multihole, small-animal single-photon emission computed tomography/computer tomography (SPECT/CT) scanner (NanoSPECT/CT, Mediso Medical Imaging Systems, Budapest, Hungary). Each head was outfitted with a tungsten-based aperture of nine 1.4 mm diameter pinholes and a thickness of 10 mm. CT scans of 7–9 min duration were followed by SPECT scans of 45–50 min. The images were acquired using Nucline software (version 10.2, Mediso Ltd., Budapest, Hungary). The real-time CT reconstruction used a cone-beam filtered backprojection. The reconstruction of SPECT data was performed with HiSPECT software (version 1.4.3049, Scivis GmbH, Göttingen, Germany) using γ-energies of 56.1 keV (±10%), 112.9 keV (±10%), and 208.4 keV (±10%) for lutetium-177. The images were prepared using VivoQuant postprocessing software (version 3.5, inviCRO Imaging Services and Software, Boston, U.S.A.) applying a Gauss postreconstruction filter (full width at half-maximum, 1.0 mm). The scale of activity was set as indicated on the images (minimum value = 0.15 Bq/voxel, maximum value = 20 Bq/voxel).

### Biodistribution Studies

The mice were injected with the radiopeptides (5 MBq, 0.5 nmol, 100 μL) in saline containing 0.05% BSA and sacrificed at 4 and 24 h after injection of the radiopeptides. Selected tissues and organs were collected, weighed, and measured with the γ-counter (1480 Wizard, PerkinElmer). The results were listed as a percentage of the injected activity per gram of tissue mass (% IA/g) using counts of a standard (defined volume of the original injection solution) measured at the same time to obtain decay-corrected values (Supporting Information). Statistical analysis was performed by applying a one-way ANOVA test with a Dunnett’s multiple comparison post-test to compare the data of the novel radiopeptides with those of [^177^Lu]­Lu-DOTA-AE105 and a Tukey’s multiple comparisons post-test to compare the novel radiopeptides among them. A *p*-value of <0.05 was considered a statistically significant difference (GraphPad Prism software, version 8.3.1).

### In Vivo Stability Studies

The ^177^Lu-labeled radiopeptides (50 MBq/nmol) were administered to immunocompetent FVB mice (25 MBq, 0.5 nmol, 100 μL), followed by collection of urine at 1 and 4 h later. Immediately after the second urine sampling, the mice were euthanized, followed by blood sampling from the heart and collection of the liver and kidneys. Due to the rapid excretion of [^177^Lu]­Lu-DOTA-AE105, this radiopeptide was also tested in additional mice that were sacrificed already at 1 h after radiopeptide injection. Blood plasma and urine samples were analyzed to determine the percentage of intact radiopeptide and the potential formation of radiometabolites using TLC as described for testing their in vitro stability (see above). Liver and kidney samples were processed as previously reported (Supporting Information)^33^ and analyzed using the same TLC system as for blood plasma and urine samples. The analysis of the thin-layer chromatograms was performed as described for the in vitro blood plasma stability experiments.

### Computational Modeling Studies

The molecular models of Lu-DOTA-AE105, Lu-uPAR-02, and Lu-uPAR-05 bound to the human uPAR were built using a template of a crystal structure (PDB ID: 1YWH) of uPAR that bound the antagonist peptide AE147 (Cha-Phe-ser-lys-Tyr-Leu-Trp-Ser-Ser-Lys; Cha: L-β-cyclohexyl-alanine; ser and lys in the D-configuration).^34^ Molecular graphics software (The PyMOL Molecular Graphics System, version 3.1.0, Schrödinger, LLC) was used to modify AE147 and generate Lu-DOTA-AE105 (Lu-DOTA-Asp-Cha-Phe-ser-arg-Tyr-Leu-Trp-Ser). To construct Lu-uPAR-02, the structure of Lu-DOTA-AE105 was modified with a lysine residue (Lu-DOTA-Lys-Asp-Cha-Phe-ser-arg-Tyr-Leu-Trp-Ser) to connect the *p*-iodophenyl-based albumin binder via a PEG_4_ spacer. Lu-uPAR-05 was constructed by modification of Lu-DOTA-AE105 with a PEG_4_ spacer between the AE105 backbone and the lysine residue that linked the *p*-iodophenyl albumin binder and the DOTA chelator. Lu-uPAR-02 and Lu-uPAR-05 were both built using the molecular modeling software Avogadro, and their geometry was optimized using the MMFF94 force field.^35^ This optimization process involved iteratively adjusting atomic positions to minimize steric clashes and ensure chemically realistic bond lengths and angles. The final peptide–protein complexes were further refined using HADDOCK software (version 2.4).^36^

## Results

### Synthesis of the uPAR-Targeting Peptides

AE105, DOTA-AE105, and the uPAR-targeting peptides designed with an albumin-binding entity were obtained in a 6–34% overall yield after 20–29 synthetic steps. The purity of all final peptides was ≥97%, as determined by HPLC analysis. The *m*/*z* ratio calculated for the respective compounds correlated well with the experimental HRMS data, confirming the chemical identity of the produced peptides (Table 1 and Figures S2–S9).

**Table 1:** Data of the Chemical Characterization of the Synthesized Peptides

compound	adduct ion	*m*/*z*_calc_	*m*/*z*_found_	yield [%]	purity [%]
AE105	[M + 2H]^2+^	635.3038	635.3045^a^	32	>99
DOTA-AE105	[M + H]^+^	1611.8166	1611.8165^b^	34	98
uPAR-01	[M + H]^+^	1006.4443	1006.4463^a^	6	>99
uPAR-02	[M + H]^+^	2259.0233	2259.0242^b^	13	97
uPAR-03	[M + H]^+^	2392.0761	2392.0760^b^	7	99
uPAR-04	[M + 3H]^3+^	713.9970	713.9952^a^	11	98
uPAR-05	[M + 2H]^2+^	1130.0153	1130.0150^a^	20	>99

a Measured by ESI-QTOF-MS.

b Measured by MALDI-TOF-MS.

### Radiolabeling and Radiolytic Stability

Radiolabeling of the uPAR-targeting peptides was performed at molar activities up to 100 MBq/nmol with a radiochemical purity of >99% determined by analytical HPLC (Figure S10). Radiolysis of all radiopeptides was almost completely prevented by the addition of l-ascorbic acid as a radical scavenger, which resulted in >93% intact radiopeptides after a 24 h incubation period (Tables 2 and S2).

**Table 2:** Summarized In Vitro Data of uPAR-Targeting Radiopeptides

	radiolytic stability after 24 h^a^	mouse blood plasma stability after 4 h^a^	human blood plasma stability after 4 h^a^	distribution coefficient^a^	uPAR-binding affinity^a^
radiopeptide	intact radiopeptide [%]	intact radiopeptide [%]	intact radiopeptide [%]	log *D* values	*K*_D_ values [nM]
[^177^Lu]Lu-DOTA-AE05	96 ± 2	13 ± 7	13 ± 6	–1.38 ± 0.18	20 ± 1
[^177^Lu]Lu-uPAR-01	93 ± 4	100 ± 0	99 ± 2	0.78 ± 0.05	48 ± 6
[^177^Lu]Lu-uPAR-02	97 ± 3	98 ± 1	99 ± 1	0.25 ± 0.02	32 ± 12
[^177^Lu]Lu-uPAR-03	98 ± 2	99 ± 1	99 ± 1	0.37 ± 0.14	56 ± 8
[^177^Lu]Lu-uPAR-04	95 ± 2	99 ± 3	98 ± 2	–0.29 ± 0.11	57 ± 21
[^177^Lu]Lu-uPAR-05	93 ± 1	98 ± 2	99 ± 1	0.24 ± 0.09	10 ± 5

a The results were calculated as the average ± SD of *n* = 3 independently performed experiments.

### Stability of the Radiopeptides in Mouse and Human Blood Plasma

The incubation of [^177^Lu]­Lu-DOTA-AE105 in mouse and human blood plasma resulted in multiple degradation products, with only ∼61 and ∼71% intact radiopeptides present after a 1 h incubation period (Tables S3 and S4). [^177^Lu]­Lu-DOTA-AE105 was further degraded, resulting in only ∼13 and ∼4.5% intact radiopeptides in both mouse and human blood plasma after 4 and 24 h of incubation, respectively. The new uPAR-targeting radiopeptides were more stable and showed >98% intact radiopeptides after a 4 h incubation period in mouse and human blood plasma (Table 2). After 24 h incubation in mouse and human blood plasma, the intact fraction of the new uPAR-targeting radiopeptides was still >98 and >90%, respectively (Tables S3 and S4).

### *n*-Octanol/PBS Distribution Coefficients

The logD value of [^177^Lu]­Lu-DOTA-AE105 was −1.38 ± 0.18, which was lower than the values obtained for the newly designed uPAR-targeting radiopeptides. Among those, [^177^Lu]­Lu-uPAR-04 was the most hydrophilic candidate, followed by [^177^Lu]­Lu-uPAR-05, [^177^Lu]­Lu-uPAR-02, and [^177^Lu]­Lu-uPAR-03. [^177^Lu]­Lu-uPAR-01, designed without additional spacer entities, was the most lipophilic radiopeptide (Table 2).

### Albumin-Binding Properties

[^177^Lu]­Lu-uPAR-01, [^177^Lu]­Lu-uPAR-02, and [^177^Lu]­Lu-uPAR-04 showed similar albumin-binding affinities in mouse and human blood plasma. [^177^Lu]­Lu-uPAR-05 showed a 1.6-fold and 2.4-fold lower albumin-binding affinity in mouse and human blood plasma than did [^177^Lu]­Lu-uPAR-01 (Figure 2A,B). [^177^Lu]­Lu-uPAR-03 showed a stronger albumin-binding affinity in mouse blood plasma. In human blood plasma, the albumin-binding affinity of [^177^Lu]­Lu-uPAR-03 was somewhat weaker than that of most other albumin-binding radiopeptides (Figure 2C,D and Table S5).

**Figure 2:**
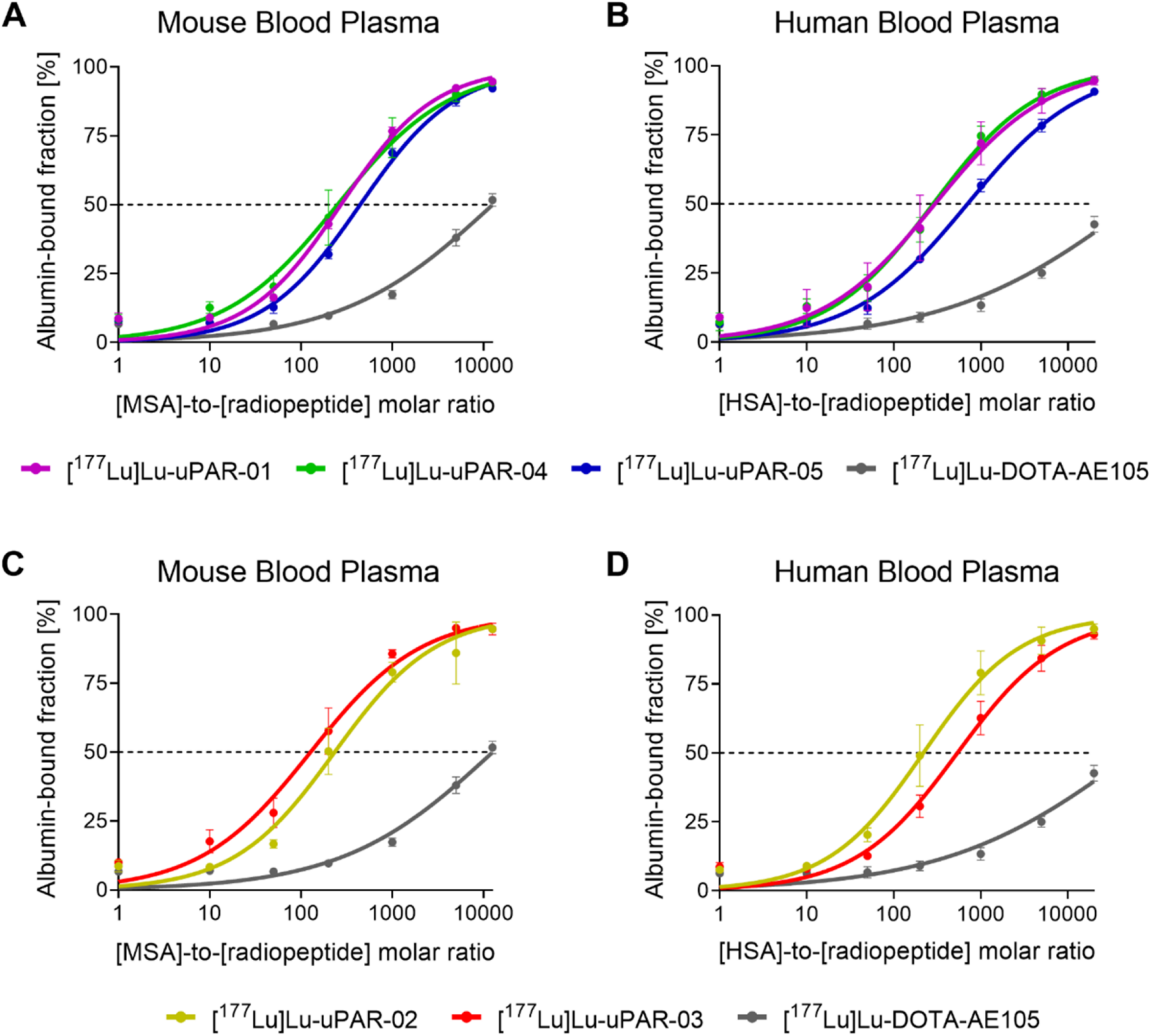
Graphs representing the albumin-binding affinity of the radiopeptides incubated with diverse dilutions of mouse and human blood plasma with defined albumin content. (A, C) Albumin-binding curves of the radiopeptides using mouse blood plasma; (B, D) albumin-binding curves of the radiopeptides using human blood plasma.

### Cell Uptake and Internalization

The HEK-uPAR cell uptake of [^177^Lu]­Lu-DOTA-AE105 reached 44 ± 4% after 2 h incubation, while the new radiopeptides modified with an albumin binder showed 21–32% uptake after the same incubation time (Figure 3). The internalized fraction of the new radiopeptides was 16–24% after 2 h, whereas the internalized fraction of [^177^Lu]­Lu-DOTA-AE105 was 11 ± 1% after 2 h. Co-incubation of uPAR with excess AE105 blocked the binding of the new radiopeptides, resulting in only 1.7–2.8% uptake after a 2 h incubation period (Table S6).

**Figure 3:**
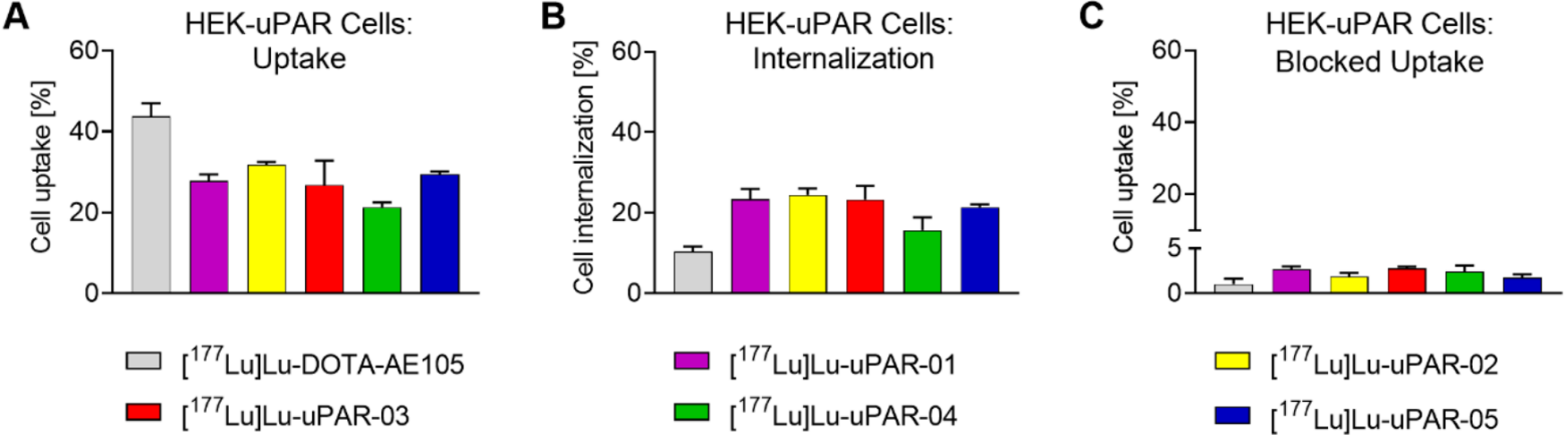
Cell uptake and internalization of the radiopeptides incubated for 2 h at 37 °C. (A) Uptake of the radiopeptides in HEK-uPAR cells; (B) cell internalized fraction of the radiopeptides; and (C) uptake in HEK-uPAR cells co-incubated with excess AE105 to block uPAR. The data are presented as average ± SD of *n* = 3 independent experiments.

### uPAR-Binding Affinity

The uPAR-binding affinity of [^177^Lu]­Lu-DOTA-AE105 was in the nanomolar range (*K*_D_ = 20 ± 1 nM) (Table 2). uPAR-binding affinities of the novel radiopeptides were similar and ranged from 10 nM to 57 nM. [^177^Lu]­Lu-uPAR-05 showed the highest uPAR-binding affinity with a *K*_D_ value of 10 ± 5 nM. The *K*_D_ values of [^177^Lu]­Lu-uPAR-04 and [^177^Lu]­Lu-uPAR-03 were somewhat higher, indicating lower uPAR-binding affinity. The binding affinities of [^177^Lu]­Lu-uPAR-01 and [^177^Lu]­Lu-uPAR-02 were about 30–40 nM.

### SPECT/CT Imaging Studies

The SPECT/CT images clearly visualized the enhanced blood retention of albumin-binding radiopeptides, in contrast to the fast blood clearance of [^177^Lu]­Lu-DOTA-AE105 (Figure 4). This was also evident from the high activity observed in the hearts of all mice injected with albumin-binding radiopeptides, in particular shortly after administration. [^177^Lu]­Lu-uPAR-03 demonstrated the longest blood circulation time, visible by the accumulation in the heart that was persistent over 24 h after injection. Over time, xenografts were visualized more effectively after injection of the albumin-binding radiopeptides, while [^177^Lu]­Lu-DOTA-AE105 accumulated more rapidly but was also effectively cleared over the first 4 h. Based on the visual assessment of the SPECT/CT images, [^177^Lu]­Lu-uPAR-02 showed the most favorable tissue distribution profile with substantial retention of activity in the xenograft and increasing xenograft-to-background contrast over time.

**Figure 4:**
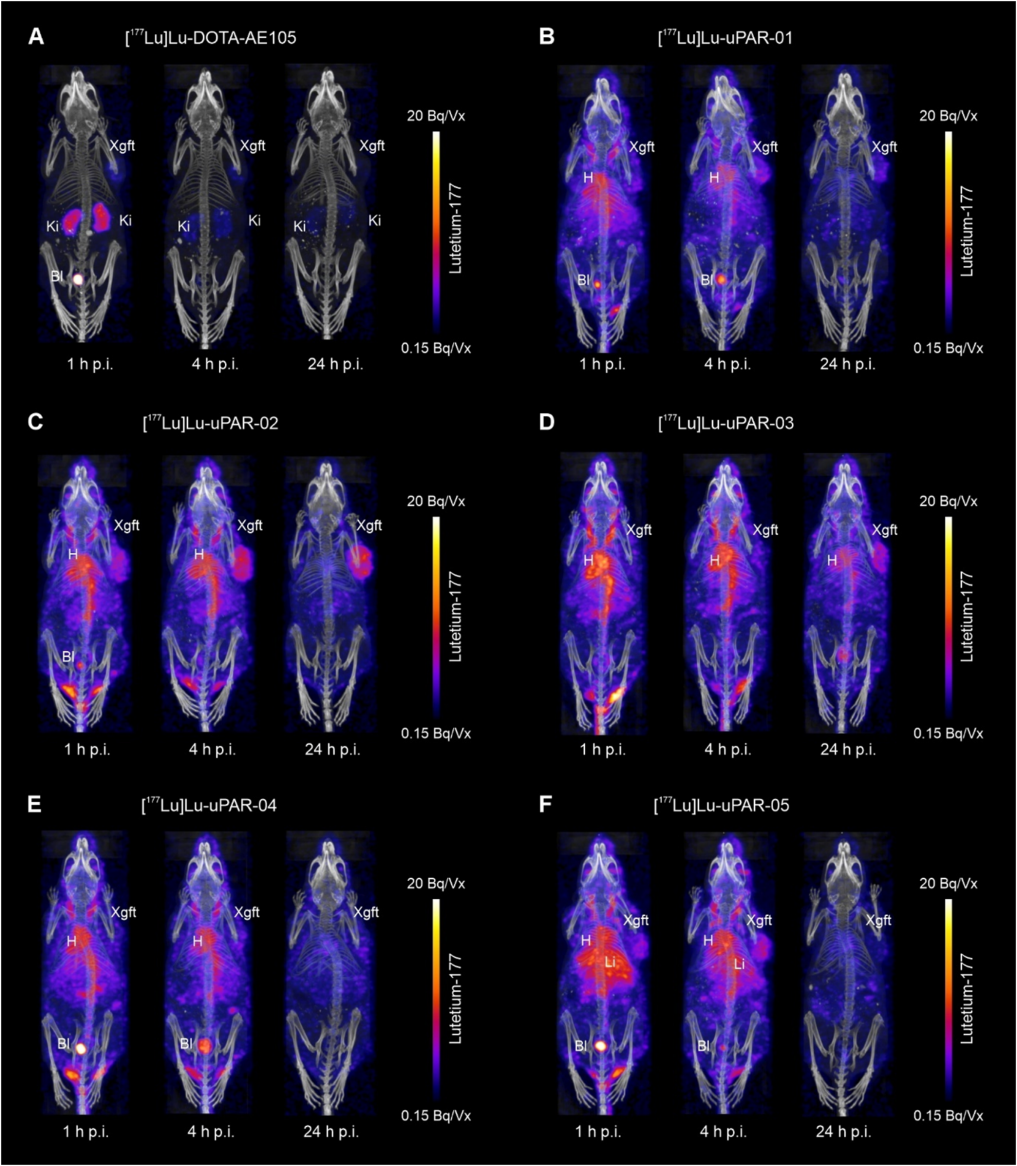
SPECT/CT imaging of HEK-uPAR xenografted mice after 1, 4, and 24 h p.i. of (A) [^177^Lu]­Lu-DOTA-AE105, (B) [^177^Lu]­Lu-uPAR-01, (C) [^177^Lu]­Lu-uPAR-02, (D) [^177^Lu]­Lu-uPAR-03, (E) [^177^Lu]­Lu-uPAR-04, and (F) [^177^Lu]­Lu-uPAR-05. Bl, urinary bladder; H, heart; Ki, kidneys; Xgft, HEK-uPAR xenograft; and Vx = voxel.

### Biodistribution Studies

Biodistribution data of [^177^Lu]­Lu-uPAR-01, [^177^Lu]­Lu-uPAR-02, [^177^Lu]­Lu-uPAR-03, [^177^Lu]­Lu-uPAR-04, and [^177^Lu]­Lu-uPAR-05 were investigated and compared with those obtained for [^177^Lu]­Lu-DOTA-AE105 (Figure 5 and Tables S7–S12). The modification with an albumin-binding entity led to significantly increased blood retention over the first 24 h of investigation (*p* < 0.05). This resulted in blood retention values of 12–16% IA/g at 4 h p.i. and 4.5–13% IA/g at 24 h after injection of the albumin-binding radiopeptides while less than 0.1% IA/g were left in blood circulation 4 h after injection of [^177^Lu]­Lu-DOTA-AE105 (Figure 5A). As a result, the accumulation of all albumin-binding radiopeptides in the HEK-uPAR xenografts (3.6–11% IA/g) was significantly higher than for [^177^Lu]­Lu-DOTA-AE105 (0.87 ± 0.05% IA/g) at 4 h p.i. (*p* < 0.05). The same held true at 24 h p.i. (4.3–10% IA/g vs 0.40 ± 0.19% IA/g, *p* < 0.05), except for [^177^Lu]­Lu-uPAR-05, which showed a higher xenograft uptake (3.1 ± 0.7% IA/g) even though not significantly different from that of [^177^Lu]­Lu-DOTA-AE105 (*p* > 0.05) (Figure 5B). Kidney accumulation of the novel radiopeptides was also significantly higher (*p* < 0.05) than for [^177^Lu]­Lu-DOTA-AE105 at both investigated time points, with the only exception of [^177^Lu]­Lu-uPAR-05, which showed lower renal retention than the other albumin-binding radiopeptides at 24 h p.i. (Figure 5C). Accumulation of the albumin-binding radiopeptides in the liver was less than 6.4% IA/g at 4 h p.i. and less than 3.2% at 24 h p.i., which was, however, significantly higher (*p* < 0.05) than the uptake found for [^177^Lu]­Lu-DOTA-AE105 (<0.2% IA/g) (Figure 5D).

**Figure 5:**
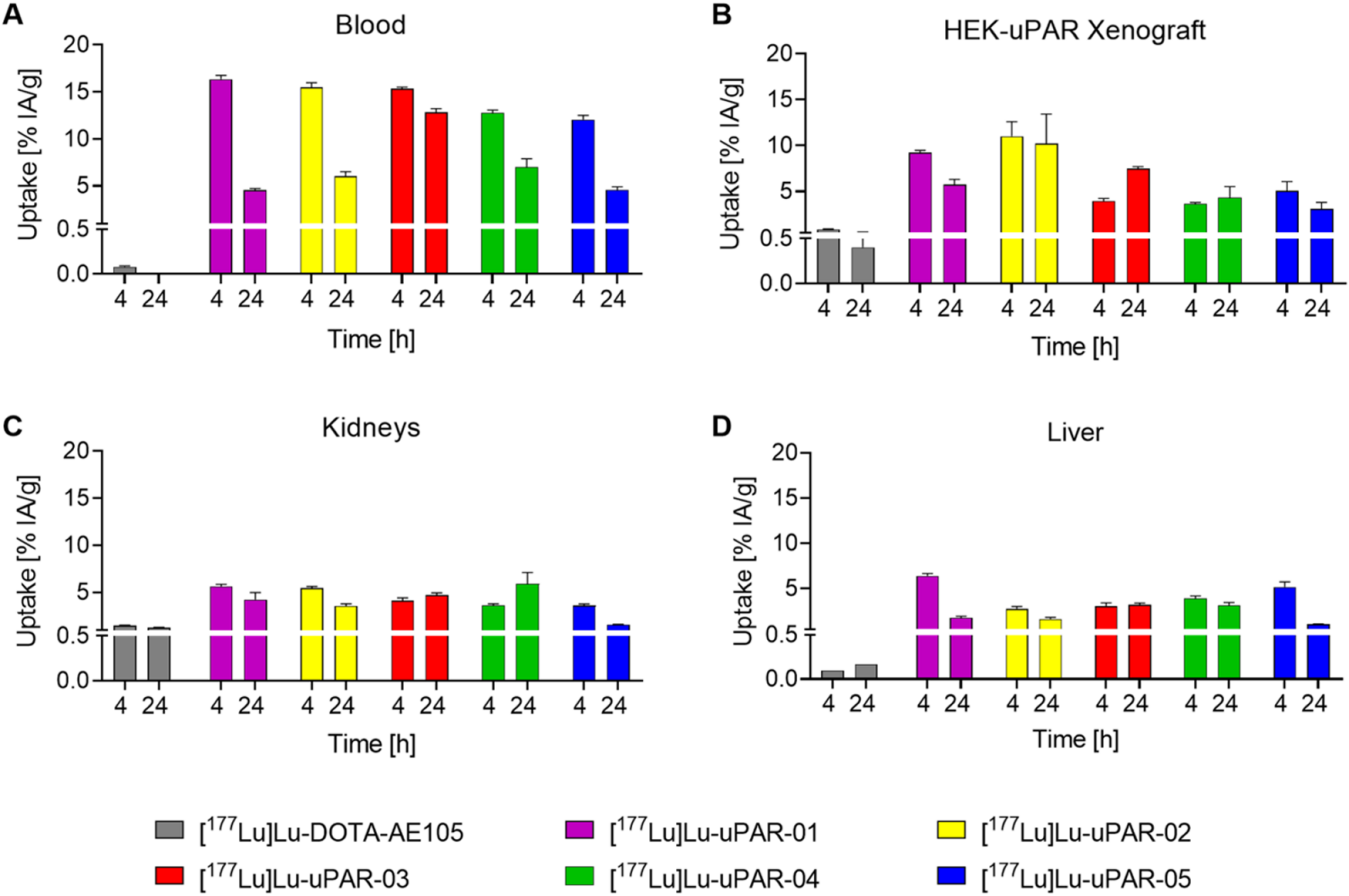
Biodistribution data of HEK-uPAR xenograft-bearing nude mice 4 and 24 h after injection of [^177^Lu]­Lu-DOTA-AE105, [^177^Lu]­Lu-uPAR-01, [^177^Lu]­Lu-uPAR-02, [^177^Lu]­Lu-uPAR-03, [^177^Lu]­Lu-uPAR-04, and [^177^Lu]­Lu-uPAR-05. (A) Uptake of the radiopeptides in blood; (B) uptake of the radiopeptides in the HEK-uPAR xenograft; (C) uptake of the radiopeptides in the kidneys; and (D) uptake of the radiopeptides in the liver.

The comparison among the albumin-binding radiopeptides revealed a favorable distribution profile for [^177^Lu]­Lu-uPAR-02. It showed the highest accumulation and retention in the HEK-uPAR xenograft (11 ± 2% IA/g; 4 h p.i. and 10 ± 3% IA/g; 24 h p.i.) among all new radiopeptides (Figure 5B). Blood clearance of [^177^Lu]­Lu-uPAR-02 was significantly faster (*p* < 0.05) than for [^177^Lu]­Lu-uPAR-03 resulting in 6.0 ± 0.5% IA/g vs 13 ± 1% IA/g activity retention at 24 h p.i. At that time point, [^177^Lu]­Lu-uPAR-01 and [^177^Lu]­Lu-uPAR-05 showed significantly lower blood activity levels (*p* < 0.05) than [^177^Lu]­Lu-uPAR-02, while [^177^Lu]­Lu-uPAR-04 showed similar blood retention as [^177^Lu]­Lu-uPAR-02 (*p* > 0.05, Figure 5A). Renal clearance of [^177^Lu]­Lu-uPAR-02 was comparable to those of the other albumin-binding radiopeptides (Figure 5C); however, retention in the liver at 4 h p.i. was significantly lower for [^177^Lu]­Lu-uPAR-02 than for all other albumin-binding radiopeptides (*p* < 0.05) except for [^177^Lu]­Lu-uPAR-03 (*p* > 0.05) (Figure 5D).

[^177^Lu]­Lu-DOTA-AE105 reached xenograft-to-blood ratios of 11 ± 1 and 32 ± 13 after 4 and 24 h, respectively, which was significantly higher (*p* < 0.05) than the analogous ratios reached with the albumin-binding radiopeptides (0.26–0.71 and 0.58–1.7 after 4 and 24 h, respectively, Figure 6A). The xenograft-to-kidney ratios were significantly higher for the albumin-binding radiopeptides than for [^177^Lu]­Lu-DOTA-AE105 (*p* < 0.05), with the only exception of [^177^Lu]­Lu-uPAR-04, which has similar ratios (*p* > 0.05) (Figure 6B). The xenograft-to-liver ratios were also significantly higher for [^177^Lu]­Lu-DOTA-AE105 than for the albumin-binding radiopeptides at 4 h p.i. (*p* < 0.05); however, at a later time point, the xenograft-to-liver ratio of [^177^Lu]­Lu-uPAR-02 was significantly higher than that of [^177^Lu]­Lu-DOTA-AE105 (*p* < 0.05) (Figure 6C). The comparison of the xenograft-to-background ratios among the albumin-binding radiopeptides revealed a favorable xenograft-to-blood ratio of [^177^Lu]­Lu-uPAR-02, which was significantly higher than for all other novel radiopeptides (*p* < 0.05) except for [^177^Lu]­Lu-uPAR-01 at 24 h p.i. (*p* > 0.05). The xenograft-to-kidney as well as the xenograft-to-liver ratios were significantly higher after injection of [^177^Lu]­Lu-uPAR-02 (*p* < 0.05) than after injection of any of the other albumin-binding radiopeptides, irrespective of the investigated time point.

**Figure 6:**
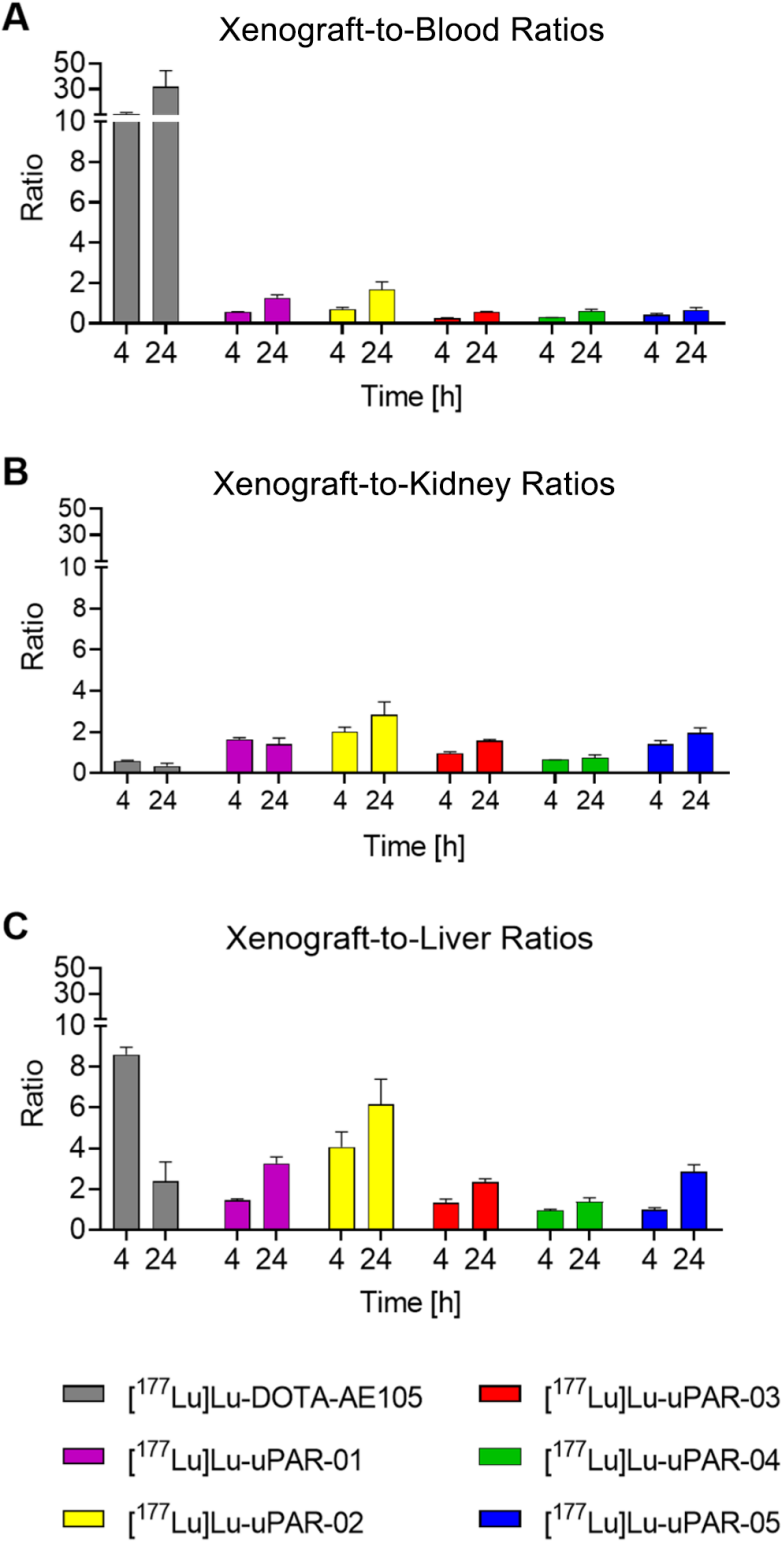
Xenograft-to-background ratios of biodistribution data of xenografted nude mice 4 and 24 h after injection of [^177^Lu]­Lu-DOTA-AE105 and ^177^Lu-labeled albumin-binding radiopeptides. (A) Xenograft-to-blood ratios, (B) xenograft-to-kidneys ratios, and (C) xenograft-to-liver ratios.

### In Vivo Stability Studies

Blood plasma, liver, kidneys, and urine samples were analyzed to assess the presence of radiometabolites following the injection of the radiopeptides. The data indicate partial in vivo metabolization of all radiopeptides; however, the albumin-binding radiopeptides were typically more stable than [^177^Lu]­Lu-DOTA-AE105. In blood plasma, [^177^Lu]­Lu-DOTA-AE105 was almost completely degraded already at 1 h after injection (Figure S11A). At 4 h after injection of [^177^Lu]­Lu-uPAR-01, [^177^Lu]­Lu-uPAR-04, and [^177^Lu]­Lu-uPAR-05, 18–30% intact radiopeptides were determined. At this same time point, ∼69 and ∼99% intact radiopeptide was determined after injection of [^177^Lu]­Lu-uPAR-02 and [^177^Lu]­Lu-uPAR-03, respectively (Table S13). A similar trend was observed in the liver and kidneys, where higher fractions of intact [^177^Lu]­Lu-uPAR-02 and [^177^Lu]­Lu-uPAR-03 were determined than for the other albumin-binding radiopeptides. The analysis of the urine samples at 1 and 4 h after injection of the radiopeptides indicated higher fractions of radiometabolites at the earlier time point, while the fraction of intact radiopeptides was, in most cases, higher at the later time point (Figures 7 and S11B).

**Figure 7:**
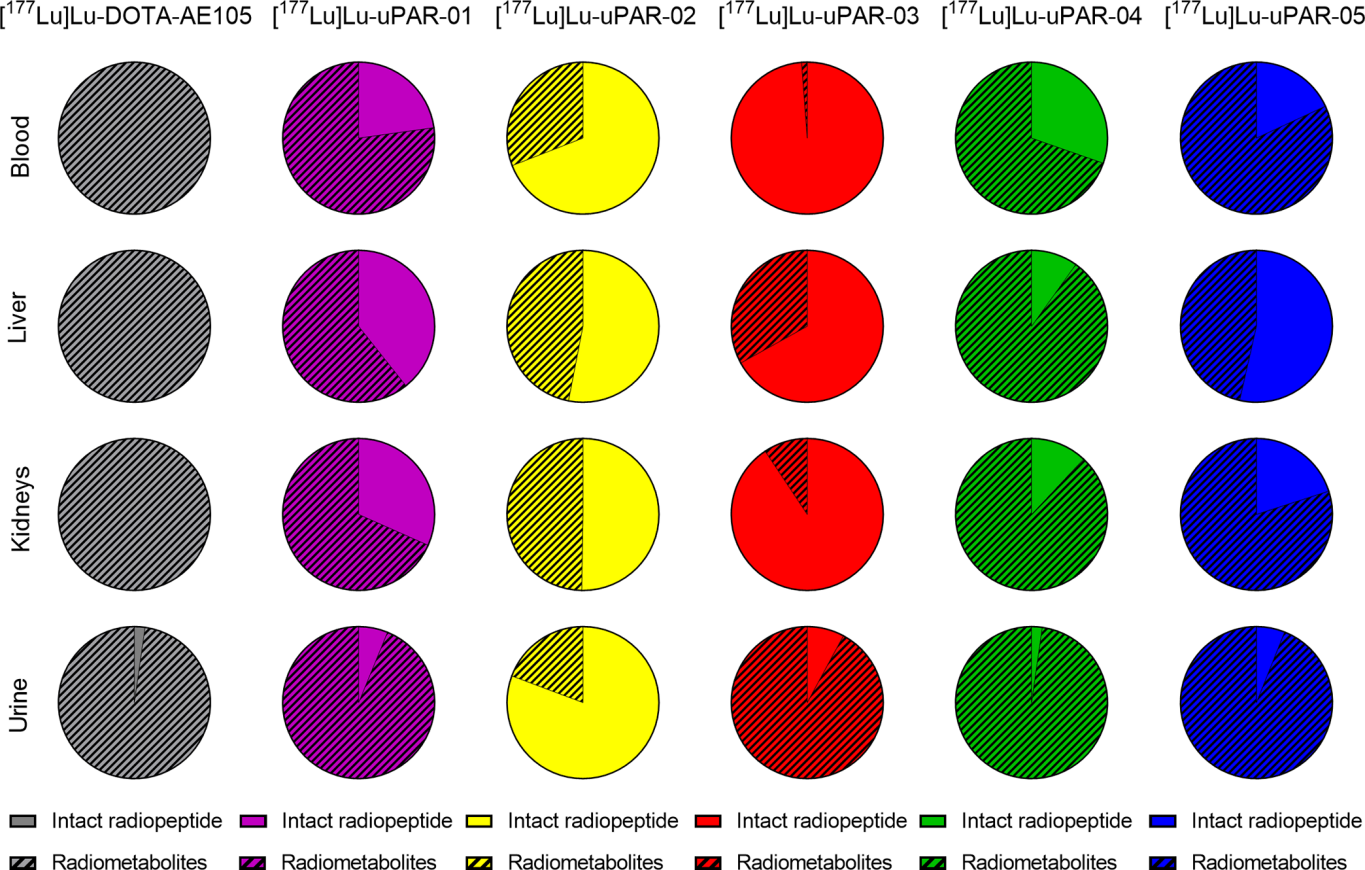
Diagrams showing the fraction of intact radiopeptides (plain color) and radiometabolites (dashed) in samples of blood plasma, liver, kidneys, and urine collected at 4 h after injection.

### Computational Modeling Studies

Molecular models of Lu-DOTA-AE105, Lu-uPAR-02, and Lu-uPAR-05 bound to uPAR (Figure 8) suggest that the AE105 peptide backbone is tightly bound to the binding pocket of uPAR while the DOTA chelator nestles within the binding site. In Lu-uPAR-02, the albumin-binding entity protrudes from the binding groove due to the PEG_4_-spacer that occupies the crevice between the N- and C-termini of the receptor and elongates the distance between the AE105 peptide backbone and the *p*-iodophenyl entity. In the case of Lu-uPAR-05, the elongation of the spacer between the AE105 peptide backbone and the DOTA chelator enables a good fit in the binding site without any possible sterical hindrance by the chelator.

**Figure 8:**
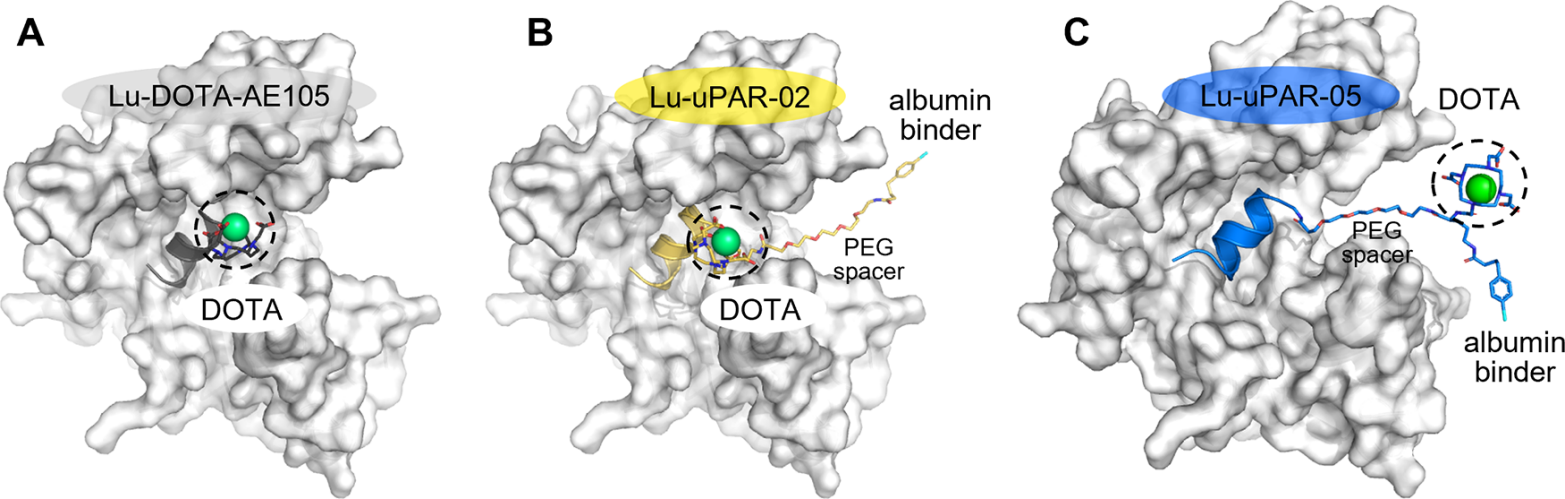
(A–C) Molecular models of three uPAR-binding radiopeptides bound to uPAR. (A) Lu-DOTA-AE105, (B) Lu-uPAR-02, and (C) Lu-uPAR-05. The peptides are depicted as gray, yellow, and blue cartoons, while the protein is shown as cartoons and a translucent molecular surface.

## Discussion

The albumin-binding uPAR-targeting peptides were synthesized using solid-phase peptide synthesis methodologies to obtain highly pure peptides, although in moderate overall yields. Radiolabeling with lutetium-177 was achieved at molar activities up to 100 MBq/nmol with >99% radiochemical purity. In line with the reported metabolic instability of [^64^Cu]­Cu-DOTA-AE105 after administration to patients,^37^ degradation of [^177^Lu]­Lu-DOTA-AE105 was observed when incubated in mouse or human blood plasma. In contrast, the new radiopeptides were more stable, demonstrated by >98% intact radiopeptides after a 4 h incubation period in mouse and human blood plasma. Obviously, the albumin-binding properties had a stabilizing effect on the radiopeptide, as previously reported for other peptidic therapeutics.^38,39^ It can be speculated that the albumin-bound fraction of the radiopeptides is resistant to enzymatic degradation by proteases, as previously demonstrated by Brandt et al., who showed that albumin-binding properties protect radioligands from neprilysine-mediated cleavage.^40^ The anticipated enhanced metabolic stability of the novel uPAR-targeting radiopeptides in mice was confirmed by the analysis of radiometabolite formation in vivo. This achievement is of critical relevance in view of employing such radiopeptides for therapeutic applications.

The novel radiopeptides showed reasonable uPAR-binding affinity with *K*_D_ values similar to that of [^177^Lu]­Lu-DOTA-AE105. Among the developed radiopeptides, [^177^Lu]­Lu-uPAR-05 exhibited an increased affinity for uPAR, possibly due to the extended spatial separation of the AE105-targeting component from the chelator and albumin binder obtained through the insertion of a PEG spacer. The introduction of the 4-(*p*-iodophenyl)­butanoate entity enabled the binding of the radiopeptides to blood plasma proteins. Relative to [^177^Lu]­Lu-DOTA-AE105, the novel radiopeptides showed a 31- to 104-fold stronger binding to mouse serum albumin and a 43- to 135-fold stronger binding to human serum albumin. The data of this study further revealed that even minor modifications next to the 4-(*p*-iodophenyl)­butanoate entity had a considerable impact on the albumin-binding affinity of the resultant radiopeptide. In particular, the introduction of an AMBA moiety, as exemplified in [^177^Lu]­Lu-uPAR-03, increased the albumin-binding affinity in mouse blood plasma, as previously seen also with folate radioconjugates designed with the same structural modifications.^23,32^ Siwowska et al. and Benešová et al. both reported on the observation that a hydrophilic spacer (e.g., PEG spacer or glutamate) next to the albumin-binding entity reduced the binding to serum albumin of folate receptor-targeting radioconjugates.^22,23^ The same was shown by Benešová et al. and Kelly et al. for PSMA radioligands.^24,26^ These findings align with the observations regarding the albumin-binding affinity of [^177^Lu]­Lu-uPAR-02, which was considerably lower due to the PEG entity next to the *p*-iodophenyl-based albumin binder. Spacer entities between the AE105 peptide backbone and the DOTA chelator, as exemplified in [^177^Lu]­Lu-uPAR-04 and [^177^Lu]­Lu-uPAR-05, did not substantially influence the overall albumin-binding properties as compared to that of [^177^Lu]­Lu-uPAR-01 without spacer entity; however, they could serve to modify the overall hydrophilicity of the radiopeptides.

As expected for any albumin-binding radiopharmaceuticals, the SPECT/CT images of mice injected with the new radiopeptides confirmed the higher background activity as a result of the enhanced blood circulation time as compared to images obtained with the fast-cleared [^177^Lu]­Lu-DOTA-AE105. As a result, the accumulation and retention of the novel radiopeptides in the HEK-uPAR xenografts were considerably higher than for [^177^Lu]­Lu-DOTA-AE105, which would be favorable in view of their therapeutic application.

Quantitative biodistribution data showed that the extensive blood circulation of [^177^Lu]­Lu-uPAR-03 resulted in slow activity accumulation in the xenograft, similar to what was previously seen with PSMA radioligands with strong albumin-binding properties.^25^ The similarly low xenograft accumulation of [^177^Lu]­Lu-uPAR-01, [^177^Lu]­Lu-uPAR-04, and [^177^Lu]­Lu-uPAR-05 may be ascribed to their moderate metabolic stability. Much more promising was [^177^Lu]­Lu-uPAR-02, presumably due to the somewhat reduced albumin-binding affinity as compared to that of [^177^Lu]­Lu-uPAR-03 but increased metabolic stability compared to the other radiopeptides. These favorable characteristics of [^177^Lu]­Lu-uPAR-02 may be the reason for its relatively fast and high accumulation in the xenograft.

Despite the favorable in vivo xenograft accumulation of [^177^Lu]­Lu-uPAR-02 as compared to that of [^177^Lu]­Lu-DOTA-AE105, the extended blood retention also resulted in a higher accumulation in healthy tissues. It remains to be investigated whether the expected increased absorbed dose in off-target tissue would cause a risk of adverse events when [^177^Lu]­Lu-uPAR-02 was applied at activities to reach therapeutic efficacy. To estimate a potential risk of bone marrow and kidney toxicity, tolerability studies should be performed using high amounts of activity.

Limitations of this study refer to the sole use of lutetium-177, which is utilized for therapeutic purposes, while the testing of the uPAR-targeting peptides with diagnostic radiometals (e.g., gallium-68) would be necessary in view of a clinical translation. A further limitation refers to the fact that the HEK-uPAR xenografted animals are an artificial model that does not precisely reflect the uPAR expression level and tumor localization found in cancer patients. Furthermore, the AE105 peptide is species-specific and does not bind to mouse uPAR;^9,10^ hence, the radiopeptide distribution in animal models may not reflect the situation as it would be expected in patients for tissues that naturally express uPAR. Studies investigating the therapeutic potential of uPAR-targeting radioligands should take these aspects into account when drawing conclusions about the tolerability of this therapy concept.

## Conclusions

The data of this study confirmed the benefit of modifying uPAR-targeting radiopeptides with an albumin-binding entity in order to achieve higher activity accumulation in the uPAR-expressing xenografts. In view of a therapeutic application, [^177^Lu]­Lu-uPAR-02, with moderate albumin-binding properties but favorable in vivo stability, was identified as the most promising candidate among the five herein investigated radiopeptides.

## Supplementary Material



## Notes

Organic synthesis of the uPAR-targeting peptides. Radiolabeling, radiolytic and in vitro stability of the uPAR-targeting peptides. Stability of the radiopeptides in mouse and human blood plasma. Determination of the distribution coefficients (log *D* values). Determination of the plasma protein-binding properties. Cell uptake and internalization studies. Determination of uPAR-binding affinity (*K*_D_ values). SPECT/CT imaging, biodistribution and in vivo metabolic stability (PDF)

## Abbreviations

AMBA: 4-(aminomethyl)­benzoic acid
BSA: bovine serum albumin
cpm: counts per minute
CT: computed tomography
DMEM: Dulbecco’s modified Eagle’s medium
DOTA: 2-[4,7,10-tris­(carboxymethyl)-1,4,7,10-tetrazacyclododec-1-yl]­acetic acid
HEK: human embryonic kidney
HPLC: high-performance liquid chromatography
HRMS: high-resolution mass spectrometry
HSA: human serum albumin
IA/g: injected activity per gram of tissue
keV: kilo-electronvolt
log *D*: logarithm of the distribution coefficient
MSA: mouse serum albumin
*m*/*z*: mass-to-charge ratio
n.c.a.: no-carrier-added
PBS: phosphate-buffered saline
PEG_4_: 3-[2-[2-[2-(2-aminoethoxy)­ethoxy]­ethoxy]­ethoxy]­propanoic acid
p.i.: post injection
PSMA: prostate-specific membrane antigen
rcf: relative centrifugal force
SD: standard deviation
SPECT: single-photon emission computed tomography
TFA: trifluoroacetic acid
TLC: thin-layer chromatography
uPAR: urokinase-type plasminogen activator receptor
