# Supplementary material for: Design and Preclinical Evaluation of Novel uPAR-Targeting Radiopeptides Modified with an Albumin-Binding Entity
Source: Mol Pharm. 2025 May 6;22(6):3242–54. doi: 10.1021/acs.molpharmaceut.5c00135 (PMC12135066; doi:10.1021/acs.molpharmaceut.5c00135)
Supplement: Supplementary file 1 [file mp5c00135_si_001.pdf]

SUPPORTING INFORMATION

Design and Preclinical Evaluation of Novel uPAR-Targeting Radiopeptides Modified with an Albumin-Binding Entity

Darja Beyer[1†], Christian Vaccarin[1†], Jerome V. Schmid[1], Luisa M. Deberle[1], Xavier Deupi[2,3,4], Roger Schibli[1, 5] and Cristina Müller[1, 5*]

[1]Center for Radiopharmaceutical Sciences, PSI Center for Life Sciences, 5232 Villigen-PSI, Switzerland

[2]Condensed Matter Theory Group, PSI Center for Scientific Computing, Theory, and Data, 5232 Villigen-PSI, Switzerland

[3]Laboratory of Biomolecular Research, PSI Center for Life Sciences, 5232 Villigen-PSI, Switzerland

[4]Swiss Institute of Bioinformatics (SIB), 1015 Lausanne, Switzerland

[5]Department of Chemistry and Applied Biosciences, ETH Zurich, 8093 Zurich, Switzerland

†Equally contributed

***Correspondence to**:
Prof. Dr. Cristina Müller
Center for Radiopharmaceutical Sciences
PSI Center for Life Sciences
Forschungsstrasse 111
5232 Villigen-PSI
Switzerland
e-mail: cristina.mueller@psi.ch
phone: +41 56 310 44 54; fax: +41 56 310 28 49

1. Organic Synthesis of the uPAR-Targeting Peptides

Purpose: AE105, DOTA-AE105 and the novel uPAR-targeting peptides (uPAR-01, uPAR-02, uPAR-03, uPAR-04 and uPAR-05; Figure S1) were synthesized using the methodology of solid phase peptide synthesis. All commercially available solvents and chemicals were used without further purification.

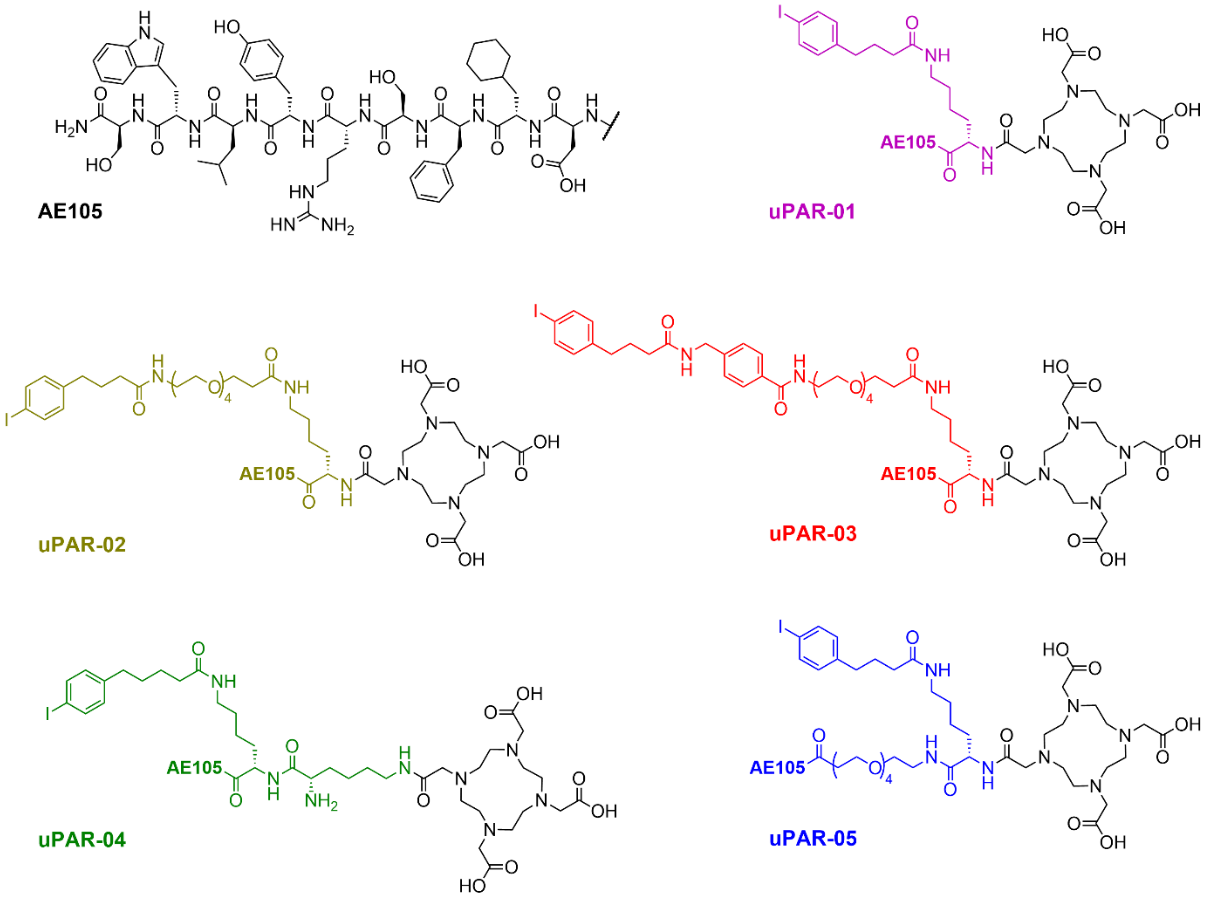

Figure S1. Overview of the chemical structures of AE105-based uPAR-targeting radiopeptides with variable linker entities.

Synthesis of resin-immobilized AE105 (RI-AE105), AE105 and DOTA-AE105. RI-AE105, AE105 and DOTA-AE105 were synthesized according to a previously reported procedure with slight modifications (Scheme S1).[1] Rink amide methylbenzhydrylamine resin (0.100 mmol, 1.00 equiv.) was weighed into a 5 mL filter-containing syringe and swelled in 3 mL anhydrous dichloromethane (DCM) for 45 min. Afterwards, the resin was conditioned with dimethylformamide (DMF) followed by Fmoc deprotection using 3 mL of a piperidine solution in DMF (50%, *v/v*) and agitated twice for 10 min. After each reaction step, the resin was washed with DMF to remove residual reagents. The resultant Fmoc-deprotected, resin-immobilized compound was added to 3 mL DMF containing Fmoc-L-Ser(^{t}Bu)-OH (0.400 mmol, 4.00 equiv.), O-(benzotriazol-1-yl)-*N,N,N',N'*-tetramethyluronium-hexafluorophosphate (HBTU, 0.396 mmol, 3.96 equiv.) and *N,N*-diisopropylethylamine (DIPEA, 0.800 mmol, 8.00 equiv) and stirred for 1 h. Conjugation of the following amino acids [Fmoc-L-Trp(Boc)-OH, Fmoc-L-Leu-OH, Fmoc-L-Tyr(^{t}Bu)-OH, Fmoc-D-Arg(Pbf)-OH, Fmoc-D-Ser(^{t}Bu)-OH, Fmoc-L-Phe-OH and Fmoc-L-Cha-OH] and Fmoc deprotection were performed according to the procedures reported above. Conjugation of the last amino acid residue, Fmoc-L-Asp(O^{t}Bu)-OH, was performed according to the described methodology, however, in this case, 1-hydroxybenzotriazole hydrate (HOBt, 0.400 mmol, 4.00 equiv.) was supplemented to the reaction mixture to suppress unwanted cyclization reactions. Fmoc deprotection of the *N*-terminus resulted in the key resin-immobilized and sidechain-protected intermediate RI-AE105. AE105 was produced by cleavage of RI-AE105 from the resin and simultaneous removal of the acid-labile protecting groups using a solution containing 95% trifluoroacetic acid (TFA) 2.5% Milli-Q water and 2.5% triisopropyl silane (TIPS) (*v/v/v*). This reaction was carried out two times for a total of 2 h, after which the volatile solvents were removed using an N_2 stream to obtain the crude AE105 peptide. For the preparation of the uPAR-targeting peptides, the following coupling steps performed on the common intermediate RI-AE105 were conducted in the presence of HOBt to suppress unwanted cyclization reactions. DOTA-AE105 was prepared by coupling of 2-[4,7,10-tris[2-[(2-methylpropan-2-yl)oxy]-2-oxoethyl]-1,4,7,10-tetrazacyclododec-1-yl]acetic acid (DOTA-tris(^{t}Bu)ester, 0.400 mmol, 4.00 equiv.) to the *N*-terminus of RI-AE105 before proceeding with the cleavage from the resin and global deprotection as described above (Scheme S1).

Scheme S1. Reaction scheme of the syntheses of RI-AE105, AE105 and DOTA-AE105. Reaction conditions: a) 50% (*v/v*) piperidine in DMF, room temperature (RT), 2 x 10 min; b) protected amino acid, HBTU, DIPEA, DMF, RT, 1 h; c) Fmoc-Asp(O^{t}Bu)-OH, HBTU, HOBt, DIPEA, DMF, RT, 1 h; d) DOTA-tris(^{t}Bu) ester, HBTU, HOBt, DIPEA, DMF, RT, 1 h **e**) TFA/TIPS/Milli-Q water, 95/2.5/2.5 (*v/v/v*), RT, 2 h.

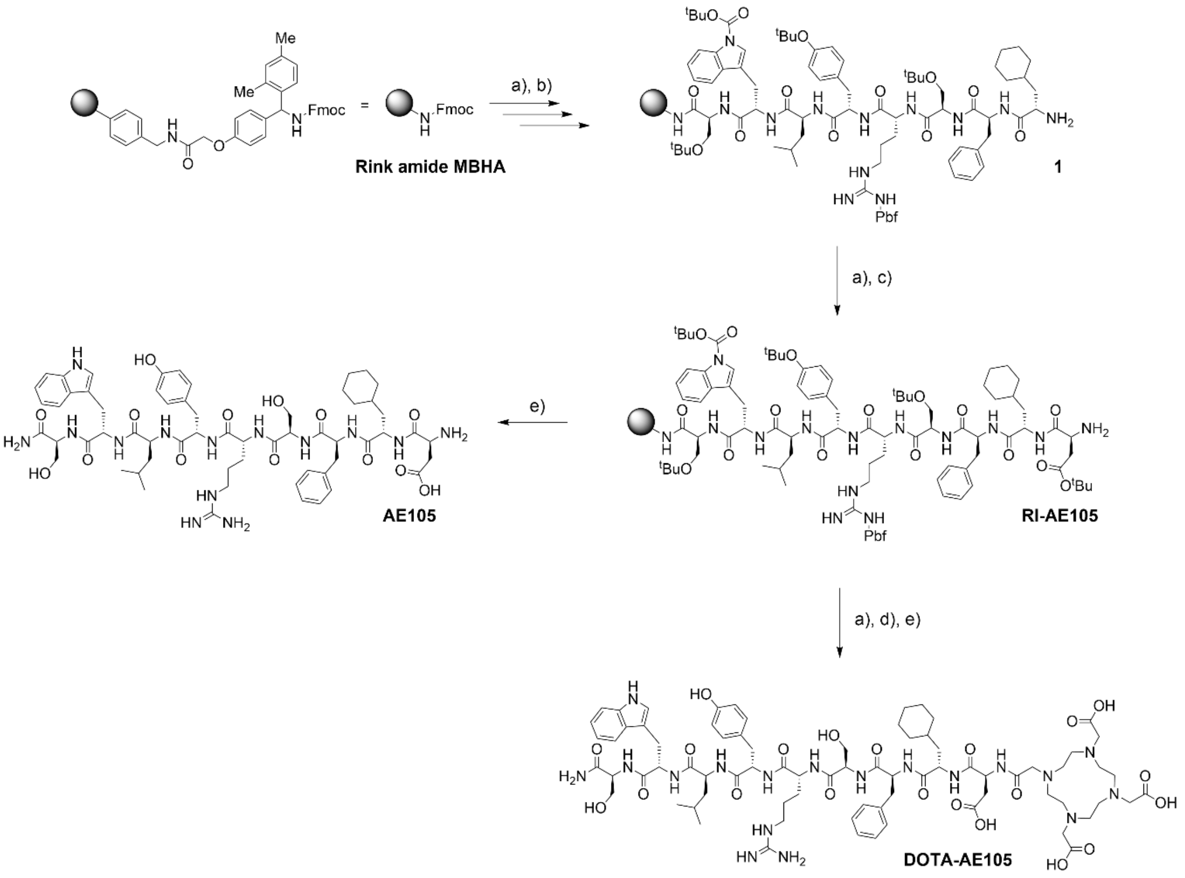

Synthesis of uPAR-01. Dde-L-Lys(Fmoc)-OH (0.400 mmol, 4.00 equiv.) was conjugated to the terminal *N*α-amino functionality of RI-AE105 (0.100 mmol, 1.00 equiv.). The Fmoc protecting group on the *N*ε-position of the inserted lysine residue was removed before conjugation of 4-(*p*-iodophenyl)butanoic acid (0.400 mmol, 4.00 equiv.). The *Nα*-1-(4,4-dimethyl-2,6-dioxocyclohexylidene)ethyl (Dde) protecting group of the lysine residue was cleaved using a solution of 2% (*v*/*v*) hydrazine hydrate in DMF (3 mL) two times for 30 min each. The resultant primary amine was conjugated with DOTA-tris(^{t}Bu)ester (0.400 mmol, 4.00 equiv.). Cleavage from the resin and general deprotection of the peptide was performed as described above, yielding uPAR-01 (Scheme S2).

Scheme S2. Synthesis scheme of uPAR-01. Reaction conditions: a) Dde-L-Lys(Fmoc)-OH, HBTU, HOBt, DIPEA, DMF, RT, 1 h; b) 50% (*v/v*) piperidine in DMF, RT, 2 x 10 min; c) 4-(*p*-iodophenyl)butanoic acid, HBTU, HOBt, DIPEA, DMF, RT, 1 h; d) 2% (*v/v*) hydrazine hydrate in DMF, RT, 2 x 30 min; e) DOTA-tris(^{t}Bu) ester, HBTU, HOBt, DIPEA, DMF, RT, 1h; f) TFA/TIPS/Milli-Q water, 95/2.5/2.5 (*v/v/v*), RT, 2 h.

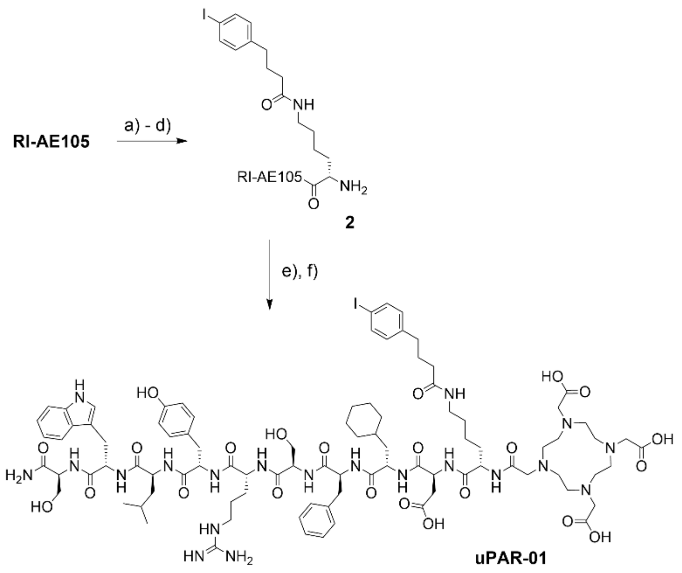

Synthesis of uPAR-02. Dde-L-Lys(Fmoc)-OH (0.4 mmol, 4.00 equiv.) was conjugated to RI-AE105 (0.100 mmol, 1.00 equiv.) following the procedure described above. The Fmoc group present on the *N*ε-position of the lysine sidechain was removed and the formed primary amino group conjugated to 3-[2-[2-[2-[2-(9H-fluoren-9-ylmethoxycarbonylamino)ethoxy]ethoxy]ethoxy]ethoxy] propanoic acid (Fmoc-PEG$_4$-OH, 0.400 mmol, 4.00 equiv.) and 4-(*p*-iodophenyl)butanoic acid (0.400 mmol, 4.00 equiv.). The Dde protecting group present at the *N*α-position of the lysine residue was cleaved using twice a mixture of 2% (*v*/*v*) hydrazine hydrate in DMF (3 mL) for 30 min. The amino group of the resin-immobilized peptide was conjugated with the DOTA-tris(^{t}Bu)ester (0.4 mmol, 4.00 equiv.). Cleavage from the resin and general deprotection of the peptide was performed as described above, yielding uPAR-02 (Scheme S3).

Scheme S3. Synthesis scheme of uPAR-01. Reaction conditions: a) Fmoc-L-Lys(Dde)-OH, HBTU, HOBt, DIPEA, DMF, RT, 1 h; b) 50% (*v/v*) piperidine in DMF, RT, 2 x 10 min; c) Fmoc-PEG$_4$-OH, HBTU, HOBt, DIPEA, DMF, RT, 1 h; d) 4-(*p*-iodophenyl)butanoic acid, HBTU, HOBt, DIPEA, DMF, RT, 1 h; e) 2% (*v/v*) hydrazine hydrate in DMF, RT, 2 x 30 min; f) DOTA-tris(^{t}Bu) ester, HBTU, HOBt, DIPEA, DMF, RT, 1 h; g) TFA/TIPS/Milli-Q water, 95/2.5/2.5 (*v/v/v*), RT, 2 h.

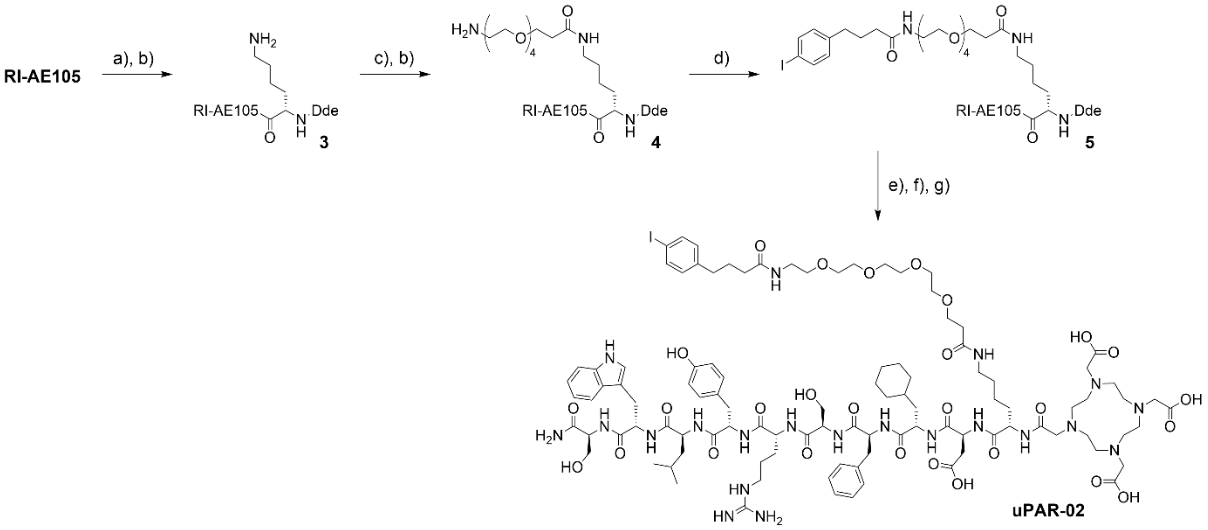

Synthesis of uPAR-03. uPAR-03 was synthesized according to the procedure used for the preparation of uPAR-02, however, a 4-[(9H-fluoren-9-ylmethoxycarbonylamino)methyl]benzoic acid (Fmoc-AMBA-OH, 0.400 mmol, 4.00 equiv.) was introduced before the conjugation of the 4-(*p*-iodophenyl)butanoic acid. Cleavage from the resin and general deprotection of the peptide was performed as described above, yielding uPAR-03 (Scheme S4).

Scheme S4. Synthesis scheme of uPAR-03. Reaction conditions: a) Fmoc-L-Lys(Dde)-OH, HBTU, HOBt, DIPEA, DMF, RT, 1 h; b) 50% (*v/v*) piperidine in DMF, RT, 2 x 10 min; c) Fmoc-PEG$_4$-OH, HBTU, HOBt, DIPEA, DMF, RT, 1 h; d) Fmoc-AMBA-OH, HBTU, HOBt, DIPEA, DMF, RT, 1 h; e) 4-(*p*-iodophenyl)butanoic acid, HBTU, HOBt, DIPEA, DMF, RT, 1 h; f) 2% (*v/v*) hydrazine hydrate in DMF, RT, 2 x 30 min; g) DOTA-tris(^{t}Bu)ester, HBTU, HOBt, DIPEA, DMF, RT, 1 h; h) TFA/TIPS/Milli-Q water, 95/2.5/2.5 (*v/v/v*), RT, 2 h.

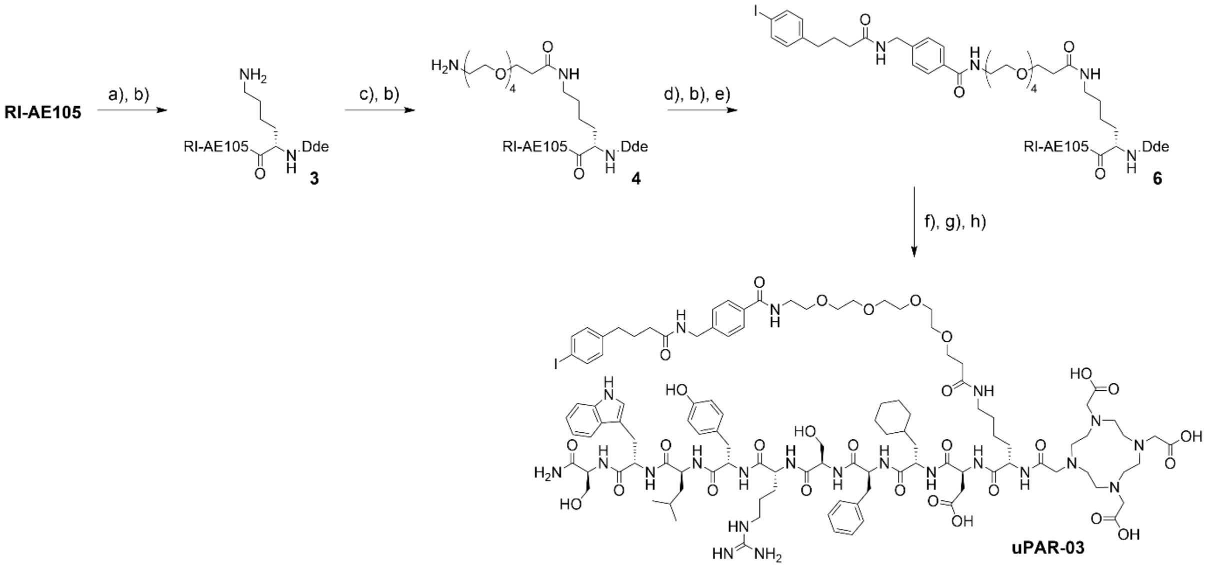

Synthesis of uPAR-04. The synthesis of uPAR-04 was performed in analogy to the procedure described for uPAR-01, but an additional lysine residue (0.400 mmol, 4.00 equiv.) was included as a spacer before the conjugation with the DOTA-tris(ᵗBu)ester (0.400 mmol, 4.00 equiv.) to its *N*ε-amino group. Subsequently, the *N*α-Dde was removed using a solution of 2% (*v*/*v*) hydrazine hydrate in DMF (3 mL) two times for 30 min each, followed by cleavage of the peptide from the resin and simultaneous removal of the acid-labile protecting groups present on the peptide and DOTA-chelator, leading to the formation of the desired uPAR-04 (Scheme S5).

Scheme S5. Synthesis scheme of uPAR-04. Reaction conditions: a) Dde-L-Lys(Fmoc)-OH, HBTU, HOBt, DIPEA, DMF, RT, 1 h; b) 50% (*v/v*) piperidine in DMF, RT, 2 x 10 min; c) 4-(*p*-iodophenyl)butanoic acid, HBTU, HOBt, DIPEA, DMF, RT, 1 h; d) 2% (*v/v*) hydrazine hydrate in DMF, RT, 2 x 30 min; e) DOTA-tris(ᵗBu)ester, HBTU, HOBt, DIPEA, DMF, RT, 1 h; f) TFA/TIPS/Milli-Q water, 95/2.5/2.5 (*v/v/v*), RT, 2 h.

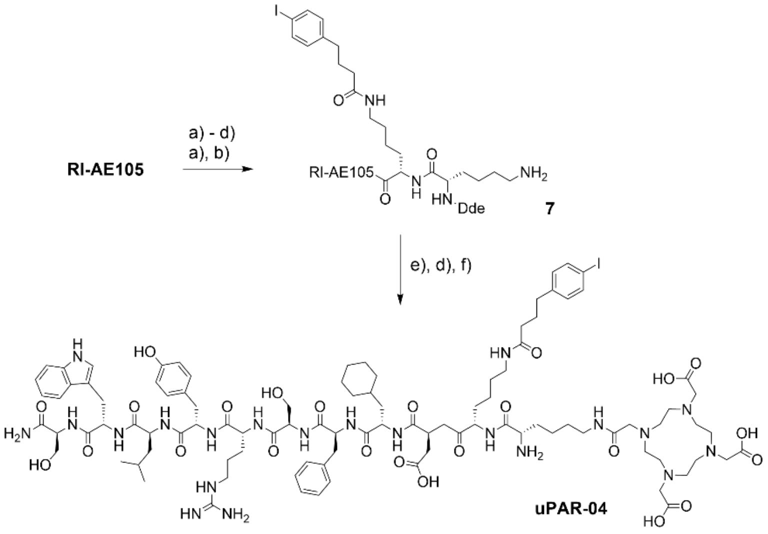

Synthesis of uPAR-05. The synthesis of uPAR-05 was performed in analogy to the procedure described for uPAR-01. In this case, Fmoc-PEG_4-OH (0.400 mmol, 4.00 equiv.) was introduced, however, as a spacer between the AE105-based targeting agent and the lysine residue that connects the 4-(*p*-iodophenyl)butanoic acid-based albumin binder and the macrocyclic DOTA chelator. Cleavage from the resin and general deprotection of the peptide was performed as described above, yielding the desired uPAR-05 (Scheme S6).

Scheme S6. Synthesis scheme of uPAR-05. Reaction conditions: a) Fmoc-PEG_4-OH, HBTU, HOBt, DIPEA, DMF, RT, 1 h; b) 50% (*v/v*) piperidine in DMF, RT, 2 x 10 min; c) Fmoc-L-Lys(Dde)-OH, HBTU, HOBt, DIPEA, DMF, RT, 1 h; d) 4-(*p*-iodophenyl)butanoic acid, HBTU, HOBt, DIPEA, DMF, RT, 1 h; e) 2% (*v/v*) hydrazine hydrate in DMF, RT, 2 x 30 min; f) DOTA-tris(^{t}Bu) ester, HBTU, HOBt, DIPEA, DMF, RT, 1 h; g) TFA/TIPS/Milli-Q water, 95/2.5/2.5 (*v/v/v*), RT, 2 h.

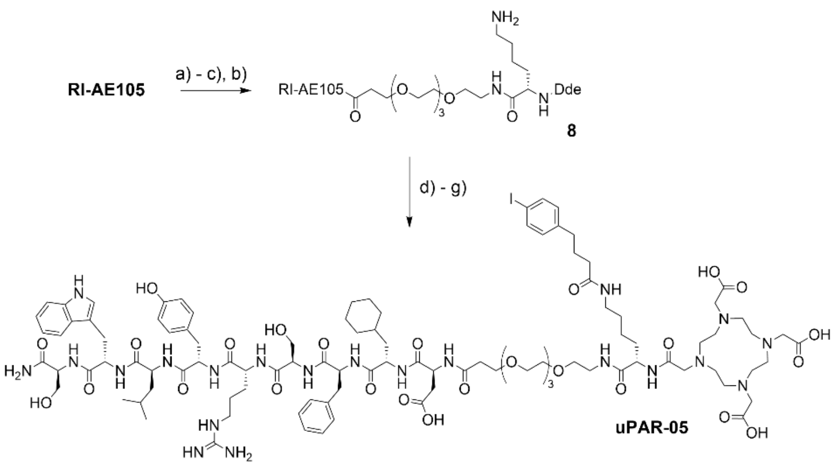

Purification of the uPAR-targeting peptides: After cleavage from the resin and general deprotection, the resulting crude products were dissolved in a 50% (*v/v*) solution of acetonitrile in Milli-Q water before purification using semipreparative high-performance liquid chromatography (HPLC) (Merck-Hitachi LaChrom HPLC system equipped with a D-7000 interface, L-7200 autosampler, L-7400 UV detector (λ=254 nm), L-7100 pump) and a reversed-phase column (C18, Sunfire™, 5 µm, 10×150 mm, Waters, Milford, MA, U.S.A.). The synthesized peptides were eluted using variable linear gradients of Milli-Q water containing 0.1% TFA (eluent A) and acetonitrile (eluent B) (Table S1). The product-containing fractions were collected in a round bottom flask, frozen in liquid N_2 and lyophilized overnight.

Table S1. HPLC Purification of the Peptides

Compound	Gradient		Flow rate	Run time	t_R
	[% A]	[% B]	[mL/min]	[min]	[min] [a]
AE105	80–58	20–42	4	15	10.3–11.0
DOTA-AE105	95–50	5–50	4	15	11.8–12.4
uPAR-01	65–55	35–45	4	15	7.20–7.90
uPAR-02	75–59	25–41	4	20	13.2–14.0
uPAR-03	75–50	25–50	4	20	14.0–14.9
uPAR-04	70–60	30–40	4	25	15.6–16.5
uPAR-05	70–55	30–45	4	25	12.0–13.3

[a] Retention time (t_R) range of the collected fractions containing the products of interest.

Characterization of the uPAR-targeting peptides. The chemical purity of the final products was determined using a reversed-phase column (C18, Sunfire™, 5 µm, 10×150 mm, Waters, Milford, MA, U.S.A.) connected to a HPLC (Merck Hitachi HPLC system, Darmstadt, Germany) equipped with a D-700 interface, a L-7200 autosampler, a L-7400 UV detector (λ= 254 nm) and a L-7100 pump. The peptides were eluted using a linear gradient of Milli-Q water containing 0.1% TFA (95–20%) and acetonitrile (5–80%) over 15 min at a flow rate of 1.0 mL/min (Table S2). The chemical identity of the final compounds was confirmed by high-resolution mass spectrometry (HRMS) analysis (ESI-QTOF-MS, Bruker maXis, Billarica, MA, U.S.A. or MALDI-TOF-MS, Bruker UltraFlex II, Billarica, MA, U.S.A.) (Table S2).

Results: The uPAR-targeting peptides were obtained in low to moderate yields of 6–34% with a high chemical purity (≥ 97%), as shown in the representative UV-Vis chromatograms below (Figure S2).

The HRMS data acquired for all the synthesized peptides correlated well with the theoretical values and are reported in the main manuscript (Table 1). The respective HRMS spectra are reported in Figure S3-S9.

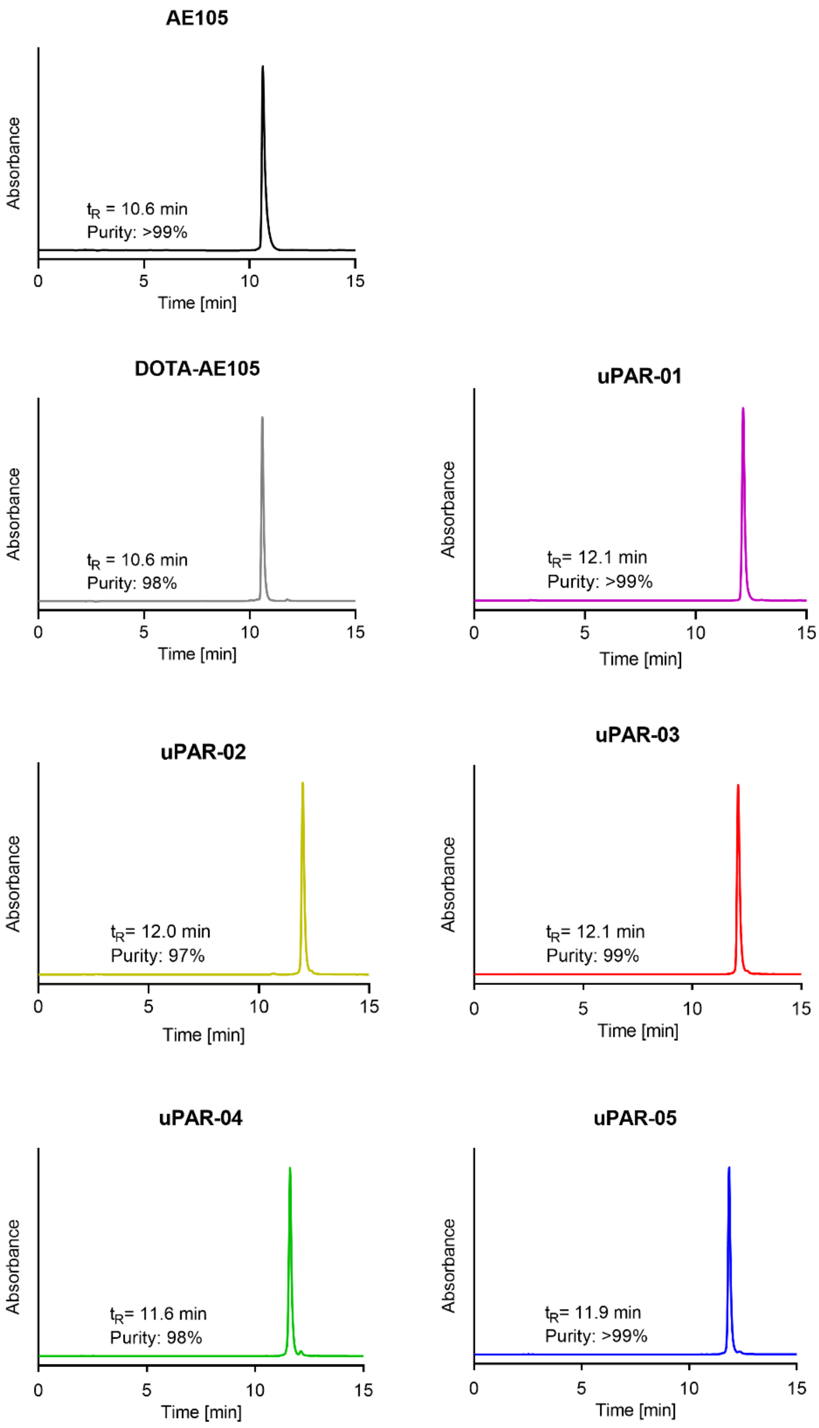

Figure S2. Representative chromatograms of the reported peptides obtained by UV-Vis HPLC (λ= 254 nm). The retention times (t_R) and chemical purities are indicated in the figure.

Compound: AE105 **Source:** ESI **Ion Polarity:** Positive

Ion Formula	Adduct	m/z	z	Meas. m/z	N-Rule	err [mDa]	err [ppm]
C60H84N14Na2O14	M+Na	635.3038	2+	635.3045	ok	0.7	1.1

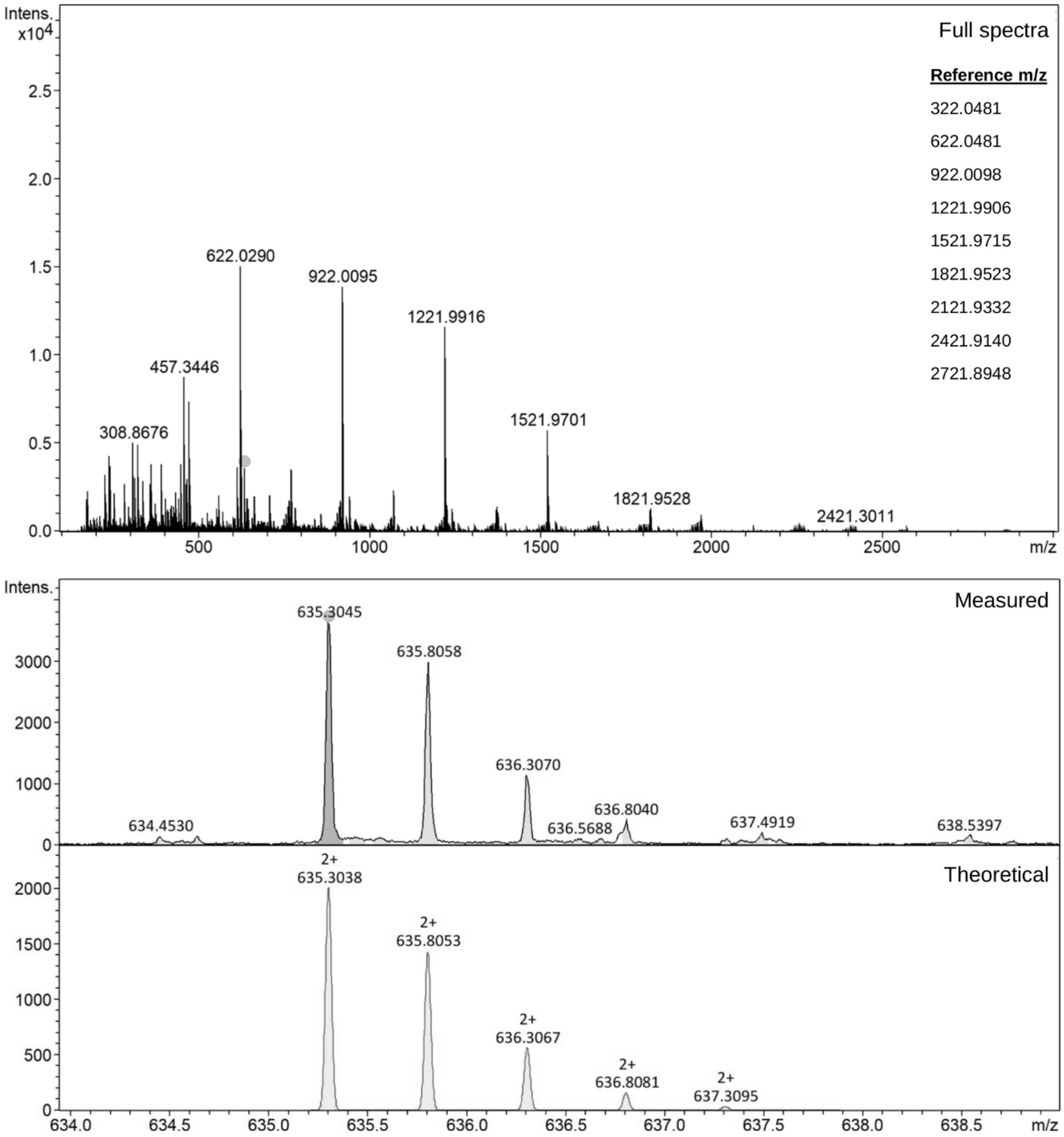

Figure S3. Full range HRMS (top) spectra of AE105 and zoom-in of the comparison between the measured spectra and the theoretical one (bottom).

Compound: DOTA-AE105 **Source:** MALDI **Ion Polarity:** Positive

Ion Formula	Adduct	m/z	z	Meas. m/z	N-Rule	err [mDa]	err [ppm]
C76H111N18O21	M+H	1611.8166	1+	1611.8165	ok	0.0	0.0

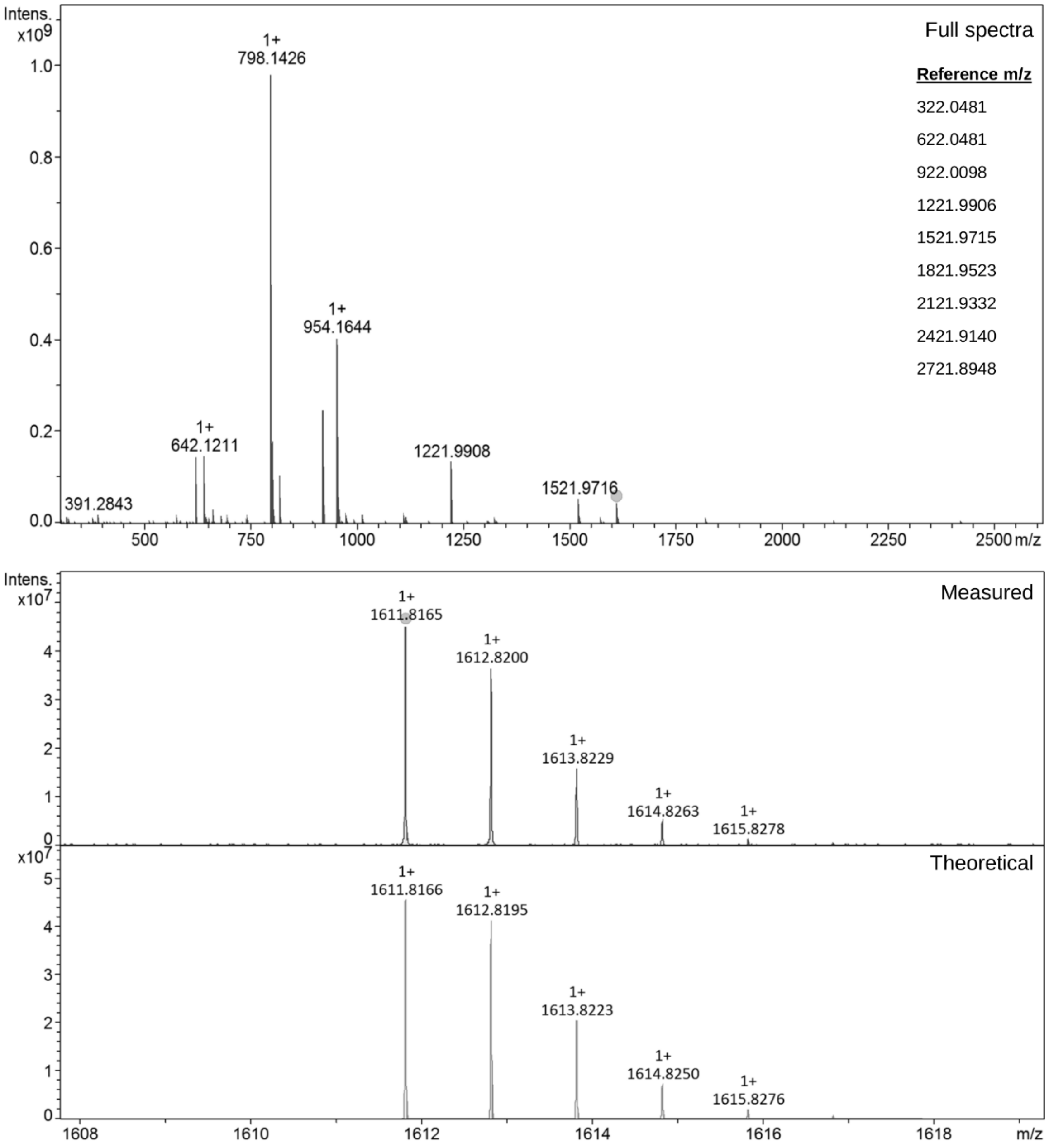

Figure S4. Full range HRMS (top) spectra of DOTA-AE105 and zoom-in of the comparison between the measured spectra and the theoretical one (bottom).

Compound: uPAR-01 **Source:** ESI **Ion Polarity:** Positive

Ion Formula	Adduct	m/z	z	Meas. m/z	N-Rule	err [mDa]	err [ppm]
C92H133IN20O23	M+H	1006.4443	2+	1006.4463	ok	-2.0	-2.0

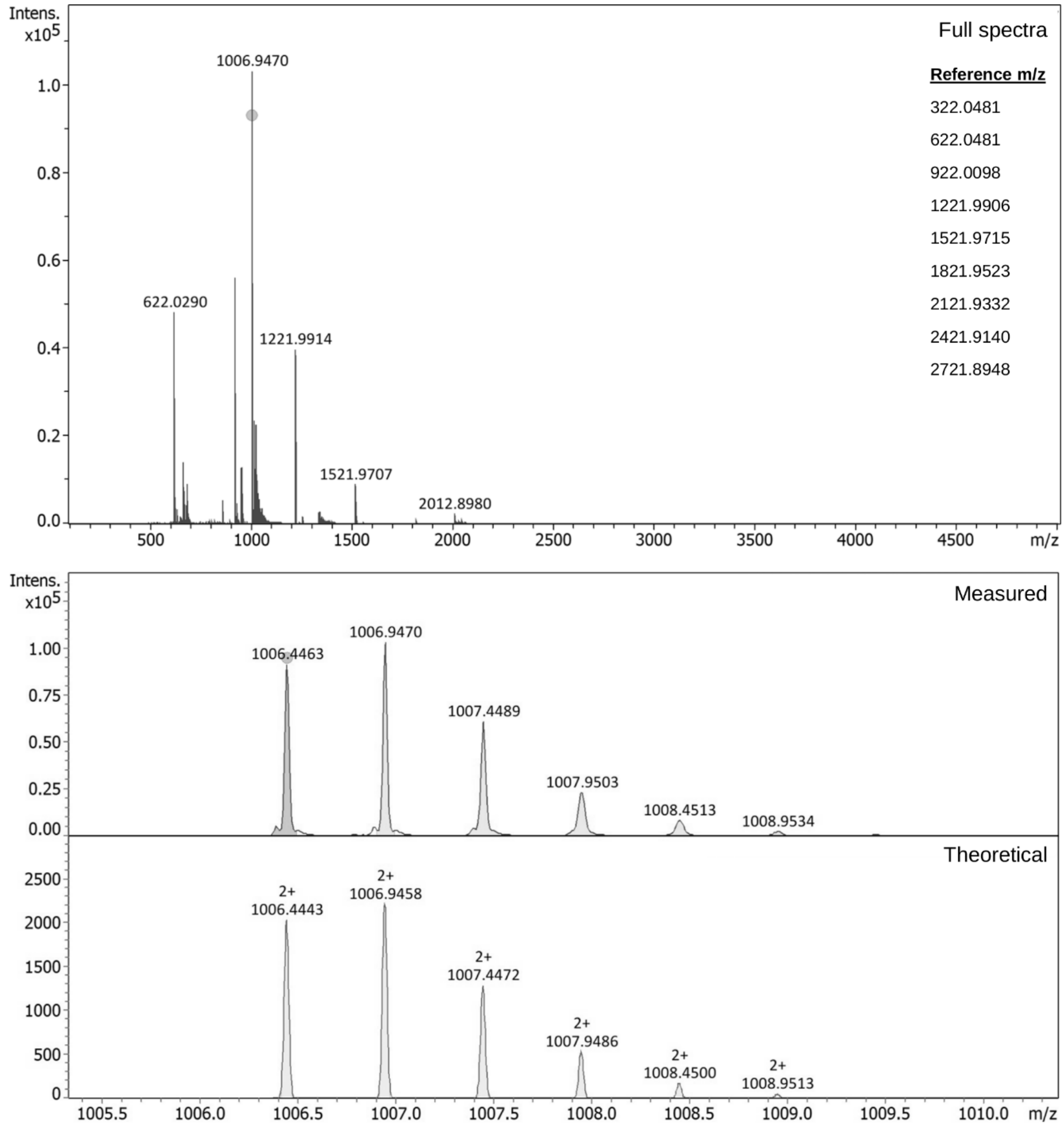

Figure S5. Full range HRMS (top) spectra of uPAR-01 and zoom-in of the comparison between the measured spectra and the theoretical one (bottom).

Compound: uPAR-02 **Source:** MALDI **Ion Polarity:** Positive

Ion Formula	Adduct	m/z	z	Meas. m/z	N-Rule	err [mDa]	err [ppm]
C103H153IN21O28	M+H	2259.0233	1+	2259.0242	ok	-0.9	-0.4

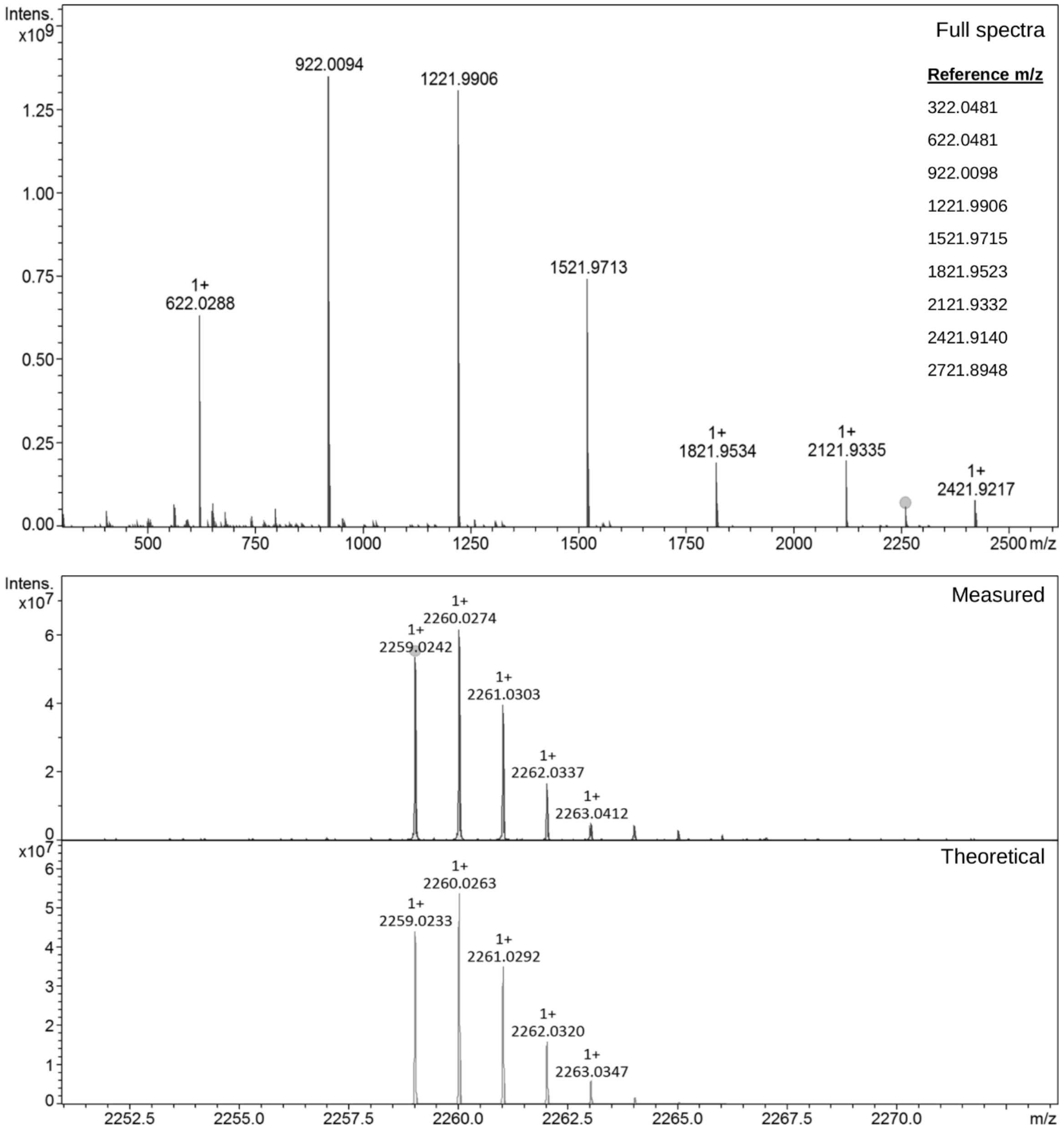

Figure S6. Full range HRMS (top) spectra of uPAR-02 and zoom-in of the comparison between the measured spectra and the theoretical one (bottom).

Compound: uPAR-03		Source: MALDI		Ion Polarity: Positive			
**Ion Formula**	**Adduct**	**m/z**	**z**	**Meas. m/z**	**N-Rule**	**err [mDa]**	**err [ppm]**
C111H160IN22O29	M+H	2392.0761	1+	2392.0760	ok	0.1	0.0

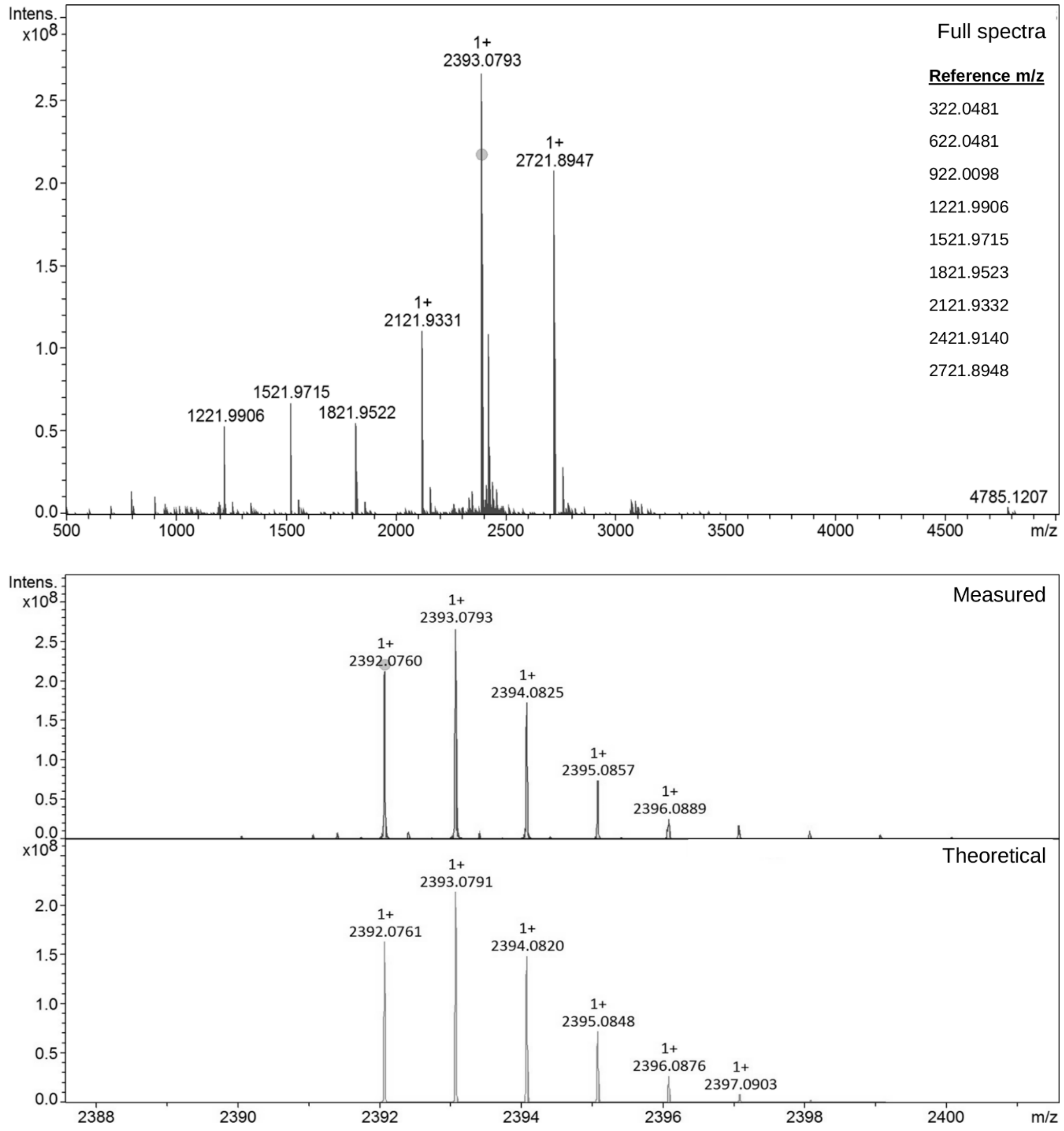

Figure S7. Full range HRMS (top) spectra of uPAR-03 and zoom-in of the comparison between the measured spectra and the theoretical one (bottom).

**Compound:** uPAR-04		**Source:** ESI		**Ion Polarity:** Positive			
**Ion Formula**	**Adduct**	**m/z**	**z**	**Meas. m/z**	**N-Rule**	**err [mDa]**	**err [ppm]**
C98H146IN22O24	M+H	713.9970	3+	713.9952	ok	1.7	2.5

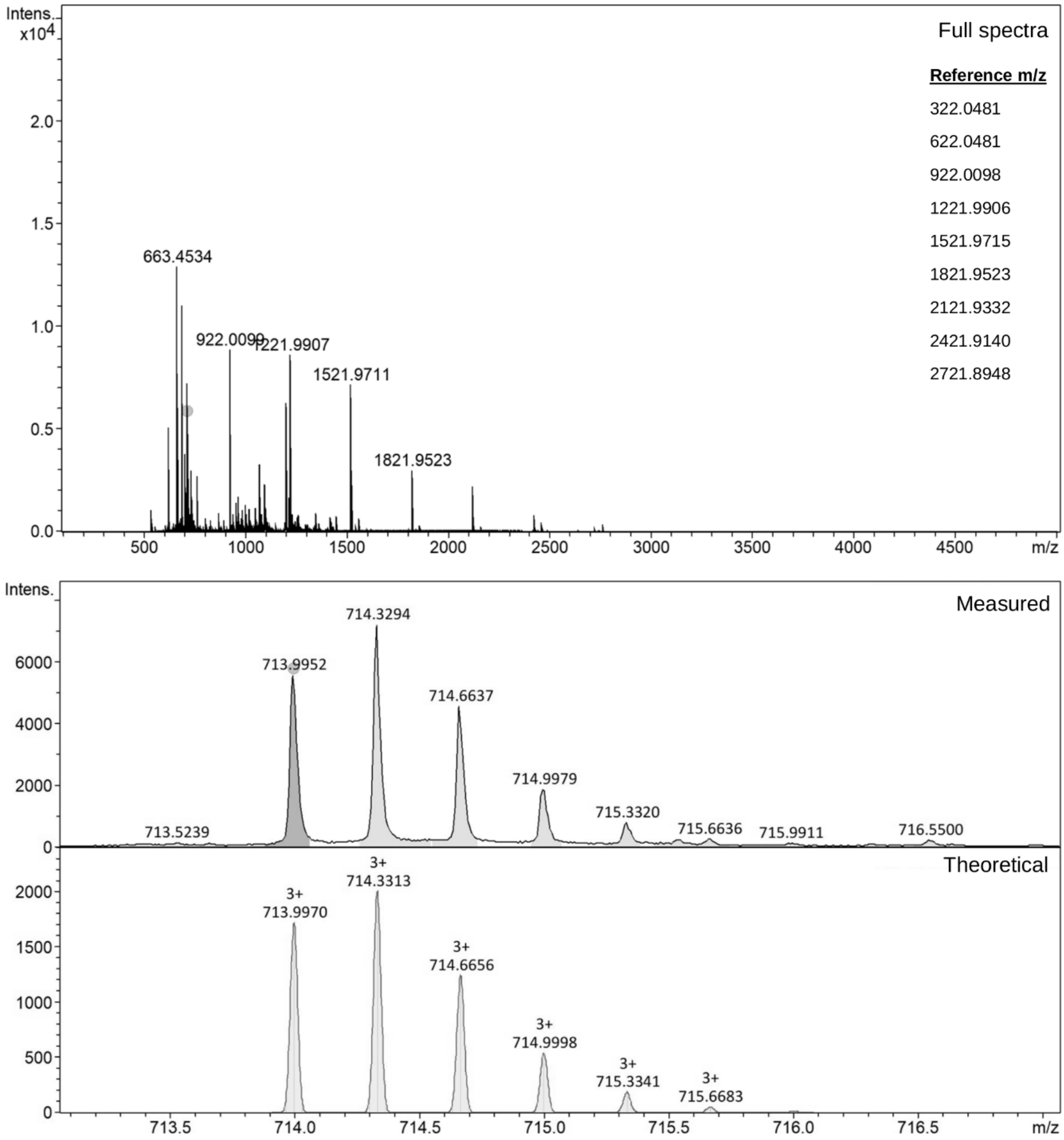

Figure S8. Full range HRMS (top) spectra of uPAR-04 and zoom-in of the comparison between the measured spectra and the theoretical one (bottom).

Compound: uPAR-05 **Source:** ESI **Ion Polarity:** Positive

Ion Formula	Adduct	m/z	z	Meas. m/z	N-Rule	err [mDa]	err [ppm]
C103H154IN21O28	M+H	1130.0153	2+	1130.0150	ok	-0.3	-0.3

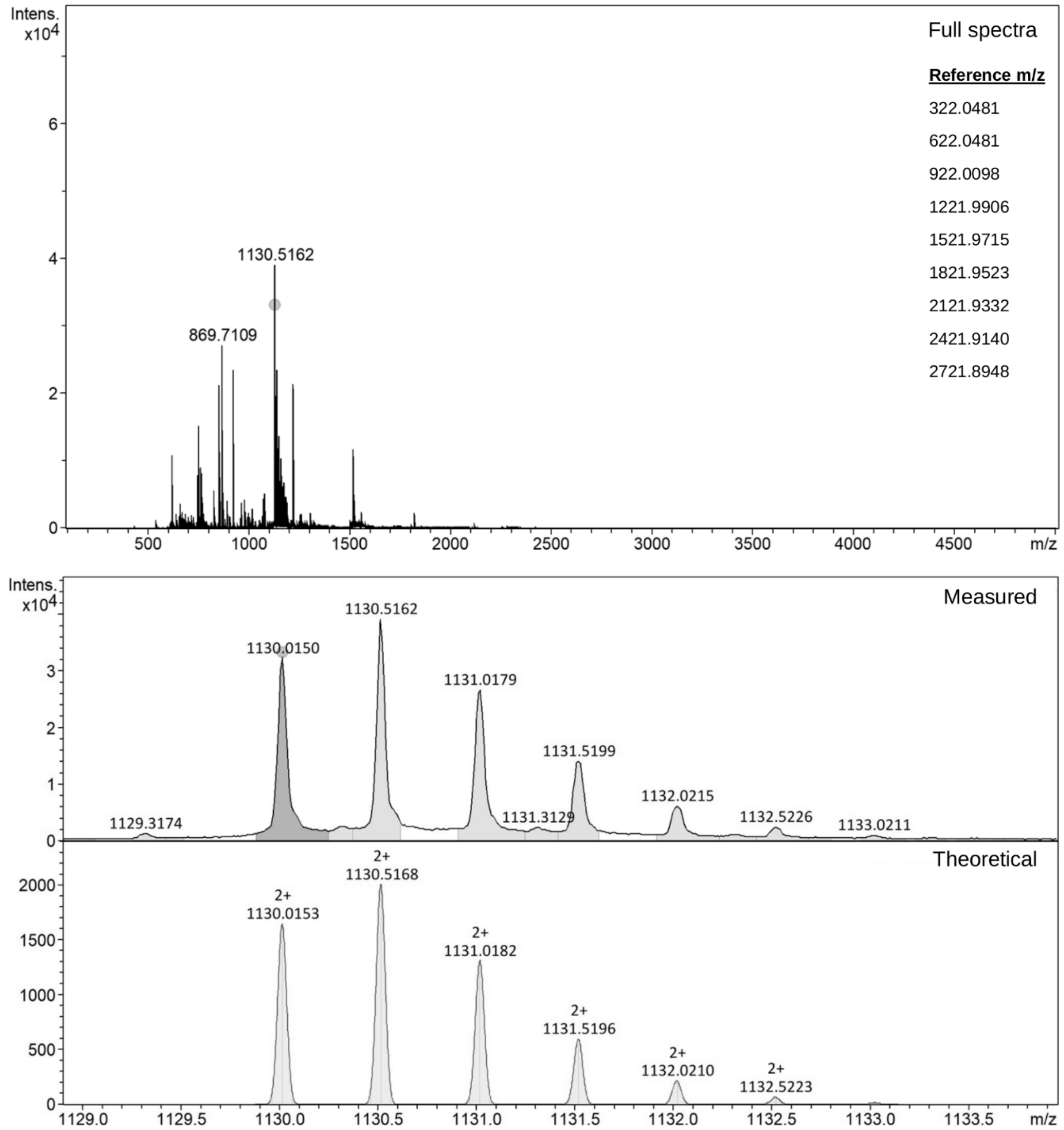

Figure S9. Full range HRMS (top) spectra of uPAR-05 and zoom-in of the comparison between the measured spectra and the theoretical one (bottom).

2. Radiolabeling and Radiolytic Stability of the uPAR-Targeting Peptides

Purpose: The new uPAR-targeting peptides were labeled with lutetium-177 and the radiolytic stability was assessed in saline.

Methods: DOTA-AE105 and the new uPAR-targeting peptides modified with an albumin binder were dissolved in Milli-Q water at a concentration of 1 mM. Only in the cases of uPAR-02 and uPAR-03, a small amount of dimethylsulfoxide (28% (*v/v*) and 36% (*v/v*), respectively), was added to facilitate the dissolution. The radiolabeling of the peptides was performed under standard labeling conditions at pH 4.5. Lutetium-177 (no-carrier-added $^{177}LuCl_3$ in 0.04 M HCl; ITM Medical Isotopes GmbH, Germany) was added to a 1/5 (*v/v*) mixture of sodium acetate (0.5 M) and HCl (0.05 M) followed by addition of the respective peptide (stock solution of 1 mM, i.e. 1 nmol corresp. 1 µL) to obtain molar activities up to 100 MBq/nmol. The respective reaction mixture was incubated for 10 min at 95 °C. Quality control of the radiolabeled peptides was performed by HPLC (Merck Hitachi HPLC system, Darmstadt, Germany, equipped with a radiodetector LB 508, Berthold Technologies) using a reversed-phase column (C18, Xterra™ MS C-18, 5 µm, 15 cm x 4.6 cm, Waters, Milford, MA, U.S.A.). The radiopeptides were eluted using a linear gradient of Milli-Q water containing 0.1% TFA (95–20%) and acetonitrile (5–80%) over 15 min at a flow rate of 1.0 mL/min.

The radiolytic stability of [^{177}Lu]Lu-DOTA-AE105 and the new ^{177}Lu-labeled uPAR-targeting radiopeptides (50 MBq/nmol) was assessed in vitro in the formulated solution. The radiopeptides were diluted in saline to obtain an activity concentration of 150 MBq in 300 µL in the presence of L-ascorbic acid (3 mg, 20 µL) and NaOAc (0.5 M, 30 µL) to compensate for the acidic pH. Aliquots of the dilutions were analyzed using HPLC after incubation periods of 1 h, 4 h and 24 h. The integrated peak area measured for the intact radioligand peak was expressed as the percentage of the sum of the integrated peak areas of each peak present in the chromatogram. The final amount of intact radiopeptide at t = 1 h, 4 h and 24 h was expressed by correcting the obtained percentage of the product peak to this peak obtained at t = 0, which was set as 100%.

Results: Radiolabeling of the peptides was performed at molar activities of up to 100 MBq/nmol with a radiochemical purity of ≥99% determined by analytical HPLC (Figure S10). In the presence of L-ascorbic acid, >93% intact radiopeptides were observed after a 24-h incubation period (Table S2).

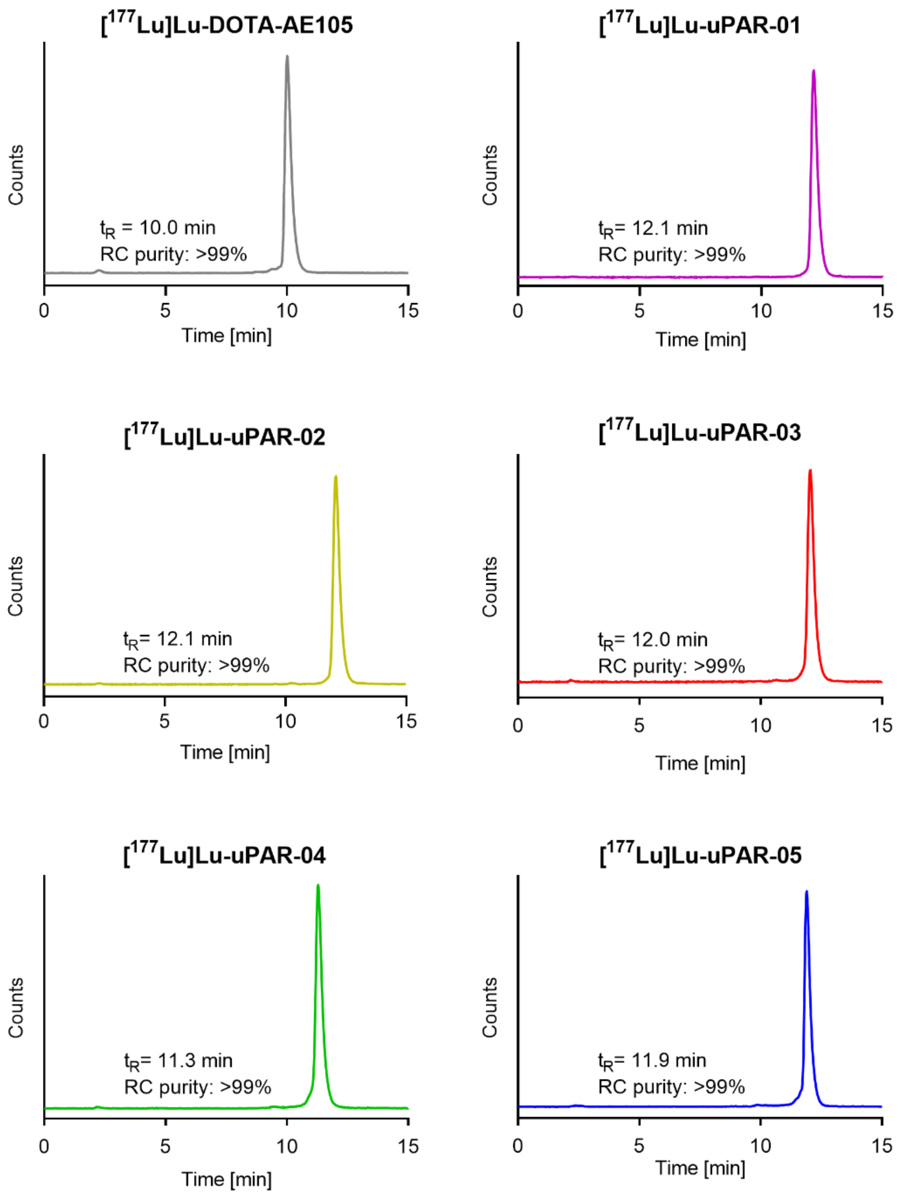

Figure S10. Representative radiochromatograms of the ^{177}Lu-labeled peptides. The retention times (t_R) and radiochemical (RC) purities are indicated in the figure.

Table S2. Stability Data of the uPAR-Targeting Radiopeptides in the Presence of L-ascorbic Acid

Radiopeptide	% Intact radiopeptide[a]		
	1 h	4 h	24 h
[^{177}Lu]Lu-DOTA-AE105	98 ± 2	98 ± 1	96 ± 2
[^{177}Lu]Lu-uPAR-01	100 ± 0	100 ± 0	93 ± 4
[^{177}Lu]Lu-uPAR-02	100 ± 1	98 ±2	97 ± 3
[^{177}Lu]Lu-uPAR-03	97 ± 3	98 ± 3	98 ± 2
[^{177}Lu]Lu-uPAR-04	99 ± 1	97 ± 2	95 ± 2
[^{177}Lu]Lu-uPAR-05	99 ± 2	97 ± 1	93 ± 1

[a]Product peak expressed as percentage of the sum of integrated peak areas of the entire chromatogram relative to the value obtained immediately after labeling (set as 100%), average ± standard deviation (SD) (n=3).

3. Stability of the Radiopeptides in Mouse and Human Blood Plasma

Purpose: The stability of the radiopeptides was assessed in mouse and human blood plasma as an indication for their in vivo stability.

Methods: The radiopeptides (50 MBq/nmol) stabilized with L-ascorbic acid were diluted in mouse blood plasma (Lot: 32321, Rockland Immunochemicals Inc., U.S.A.) or in human blood plasma (Stiftung Blutspende SRK Aargau-Solothurn, Switzerland) at a concentration of 10 MBq/200 µL and incubated at 37 °C over a 24 h time period. Control samples were prepared by diluting the radiopeptides in saline at the same activity concentration. Aliquots of each dilution were taken after 1 h, 4 h, and 24 h (2 µL, ~100 kBq) and investigated employing thin layer chromatography (TLC) methods on reversed-phase TLC plates (MERCK Analytical Chromatography, TLC Silica gel 60 RP-18 F_{254}s) as stationary phase and a 3/7 (*v*/*v*) mixture of acetonitrile/citrate buffer (0.1 M, pH 5.5) as mobile phase. A dilution of [^{177}Lu]$LuCl_3$/HCl 0.04 M (10 MBq/200 µL) was added on each TLC plate. The developed and dried TLC plates were exposed to a phosphor screen (Super resolution screen PSR10450013, PerkinElmer) for 1 min. The screen was read with a Cyclone Plus phosphor imager (PerkinElmer) and the peak quantification was performed using OptiQuant Software (version 5.0).

Results: The results are reported in the main article and in Table S3–S4.

Table S3. Stability Data of uPAR-Targeting Radiopeptides Incubated in Mouse Blood Plasma

Radiopeptide	Intact radiopeptide in mouse blood plasma [%][a]		
	1 h	4 h	24 h
[^{177}Lu]Lu-DOTA-AE105	61 ± 6	13 ± 7	4.6 ± 2.2
[^{177}Lu]Lu-uPAR-01	100 ± 0	100 ± 0	99 ± 1
[^{177}Lu]Lu-uPAR-02	100 ± 1	98 ± 1	98 ± 2
[^{177}Lu]Lu-uPAR-03	100 ± 0	99 ± 1	98 ± 1
[^{177}Lu]Lu-uPAR-04	101 ± 2	99 ± 3	98 ± 0
[^{177}Lu]Lu-uPAR-05	99 ± 1	98 ± 2	98 ± 2

[a]Product peak expressed as percentage of the sum of integrated peak areas of the entire TLC chromatogram relative to the value obtained immediately after labeling (set as 100%), average ± SD (n=3).

Table S4. Stability Data of uPAR-Targeting Radiopeptides Incubated in Human Blood Plasma

Radiopeptide	Intact radiopeptide in human blood plasma [%][a]		
	1 h	4 h	24 h
[^{177}Lu]Lu-DOTA-AE105	71 ± 3	13 ± 6	4.5 ± 1.2
[^{177}Lu]Lu-uPAR-01	100 ± 0	99 ± 2	99 ± 1
[^{177}Lu]Lu-uPAR-02	100 ± 1	99 ± 1	99 ± 1
[^{177}Lu]Lu-uPAR-03	100 ± 1	99 ± 1	99 ± 2
[^{177}Lu]Lu-uPAR-04	101 ± 3	98 ± 2	90 ± 5
[^{177}Lu]Lu-uPAR-05	99 ± 1	99 ± 1	99 ± 1

[a]Product peak expressed as percentage of the sum of integrated peak areas of the entire TLC chromatogram relative to the value obtained immediately after labeling (set as 100%), average ± SD (n=3).

4. Determination of the *n*-Octanol/PBS Distribution Coefficients (logD Values)

Purpose: The distribution coefficients were determined as a measure for the hydrophilic/lipophilic character of the respective radiopeptide.

Methods: The logD value of all radiopeptides was determined in a mixture of *n*-octanol and phosphate buffered saline (PBS) pH 7.4 using phase separation followed by liquid-liquid extraction. Five polystyrene radioimmunoassay (RIA) tubes were prepared for each radiopeptide with 1.5 mL *n*-octanol and 1.475 mL PBS pH 7.4. The RIA tubes were rigorously vortexed and subsequently let to stand so that the phase separation could take place. The radiopeptides (50 MBq/nmol) were diluted with PBS pH 7.4 to obtain an activity concentration of 10 MBq/0.5 mL. A volume of 25 µL of these solutions was added to each RIA tube (25 µL, ~0.5 MBq, 0.01 nmol) followed by vortexing for 1 min. Phase separation was achieved by centrifugation at 560 rcf for 6 min. The activity in the *n*-octanol and PBS phase, respectively, was measured in a γ-counter (Wallac Wizard 1480, PerkinElmer). The distribution

coefficients were calculated as the logarithm of the ratio of counts per minute (cpm) measured in the *n*-octanol phase relative to the cpm measured in the PBS pH 7.4 phase. Each experiment was performed as described above three independent times for each radiopeptide.

Results: The results are reported in the main article.

5. Determination of the Albumin-Binding Properties

Purpose: The radiopeptides were investigated with regard to their albumin-binding capabilities in vitro as a measure to predict blood retention in vivo.

Methods: The relative albumin-binding affinity of the radiopeptides in mouse blood plasma (Rockland Immunochemicals, Inc., U.S.A.) and human blood plasma (Stiftung Blutspende SRK Aargau-Solothurn, Switzerland) were determined using an ultrafiltration method. The amount of mouse serum albumin (MSA) and human serum albumin (HSA) in mouse and human blood plasma was defined as 550 μM and 800 μM, respectively, based on measurements using a dry chemistry analyzer (DRI-CHEM 4000i, FUJIFILM, Japan). A fixed amount of radiopeptide (50 MBq/nmol, ~300 kBq, 15 μL, 0.006 nmol) was added to a defined volume (150 μL) of mouse and human blood plasma and various dilutions thereof in PBS pH 7.4, resulting in defined [MSA]-to-[radiopeptide] or [HSA]-to-[radiopeptide] molar concentration ratios ranging from 0.01–12500 and 0.01–20000, respectively, followed by incubation of the samples at 37 °C for 30 min. Ice-cold PBS (150 µL, pH 7.4) was added before loading the blood plasma samples on Amicon centrifugal filters (cut-off of 10 kDa; Merck Millipore) followed by centrifugation (14000 rcf, 30 min, 4 °C) to allow the separation of the plasma protein-bound from the plasma-unbound (free) fractions of each sample. The inserts of the filter devices were inverted and centrifuged at 200 rcf for 3 min to recover the protein-bound radiopeptide. The activity in the filtrate and filter unit was measured in a γ-counter (1480 Wizard, PerkinElmer) and the counts were combined assuming that the fraction retained in the filter membrane was not bound to proteins. The protein-bound fraction was expressed as percentage of the whole activity (i.e. plasma protein-bound activity, activity measured in the filtrate and activity measured in the filter (set as 100%)). The data were analyzed using a semi-logarithmic plot assuming a maximum binding of 100%. The Hill equation was fitted to the data points and the half-maximum binding (B_{50}) based on the average curve obtained from three independent experiments (GraphPad Prism software, version 8.3.1). The relative albumin-binding affinities of the radiopeptides were defined as the inverse ratio of the B_{50} value using [^{177}Lu]Lu-uPAR-01 as the reference compound, whose relative binding affinity was set as 1.0. The results were presented as the B_{50} value obtained from the average curve from 3 independent experiments.

Results: The results are reported in the main article and in Table S5.

Table S5. Relative Albumin-Binding Affinities of the Radiopeptides in Mouse and Human Blood Plasma

Radiopeptide	Mouse blood plasma	Human blood plasma
[^{177}Lu]Lu-DOTA-AE05	0.02	0.01
[^{177}Lu]Lu-uPAR-01	1.00	1.00
[^{177}Lu]Lu-uPAR-02	1.17	1.36
[^{177}Lu]Lu-uPAR-03	2.08	0.56
[^{177}Lu]Lu-uPAR-04	1.08	1.07
[^{177}Lu]Lu-uPAR-05	0.61	0.43

[a]Normalized to the affinity of [^{177}Lu]Lu-uPAR-01 set as 1.00.

6. Cell Uptake and Internalization Studies

Purpose: The uptake and internalization of the uPAR-targeting radiopeptides were investigated using HEK cells transfected with human uPAR (HEK-uPAR cells) as a model cell line.

Methods: HEK-uPAR cells were seeded in poly-D-lysine-coated 12-well plates (1×10^6 cells in 2 mL per well) using Dulbecco's Modified Eagle Medium (DMEM) cell culture medium with supplements. The cells were incubated at 37 °C and 5% CO_2 overnight to allow cell adhesion and growth. After removal of the supernatant, the HEK-uPAR cells were rinsed with PBS before adding DMEM without supplements (975 µL per well). The radiopeptides (50 MBq/nmol) were diluted in saline comprising 0.05% bovine serum albumin and added to each well in a volume of 25 µL (0.75 pmol, 38 kBq). In some wells, the HEK-uPAR cells were coincubated with an excess of AE105 (final concentration of 5 µM) to block uPAR. After incubation of the cells for 2 h or 4 h at 37 °C, they were rinsed with ice-cold PBS to determine the total uptake of the radiopeptides. In order to assess the internalized fraction, an acidic glycine buffer (pH 2.8, 50 mM glycine, 100 mM NaCl) was applied to release uPAR-bound radiopeptides from the cell surface and, hence, determine the internalized fraction only. The cells were lysed using an aqueous NaOH solution (1 M, 1 mL) and the lysates were transferred to RIA tubes for counting the activity in a γ-counter (1480 Wizard, PerkinElmer). The protein concentration of each sample was determined using a Micro BCA Protein Assay kit (Pierce, Thermo Scientific) to standardize the measured activity to the average content of protein in a single well. The uptake and internalized fraction were expressed as the percentage of total added activity and presented as the average ± SD of n = 3 independent experiments.

Results: The results are reported in the main article and Table S6.

Table S6. Data of Cell Uptake and Internalization of the Radiopeptides

Radiopeptide	Uptake [%]		Internalization [%]		Blocked uptake [%]	
	2 h	4 h	2 h	4 h	2 h	4 h
[^{177}Lu]Lu-DOTA-AE05	44 ± 4	46 ± 5	11 ± 1	17 ± 3	1.0 ± 0.6	1.6 ± 1.0
[^{177}Lu]Lu-uPAR-01	28 ± 2	24 ± 5	24 ± 3	21 ± 2	2.8 ± 0.6	3.2 ± 0.4
[^{177}Lu]Lu-uPAR-02	32 ± 1	31 ± 1	24 ± 2	25 ± 1	1.8 ± 0.4	1.9 ± 0.3
[^{177}Lu]Lu-uPAR-03	27 ± 6	25 ± 4	23 ± 4	21 ± 3	2.8 ± 0.2	3.0 ± 0.1
[^{177}Lu]Lu-uPAR-04	21 ± 1	18 ± 1	16 ± 3	12 ± 2	2.4 ± 0.7	2.6 ± 0.5
[^{177}Lu]Lu-uPAR-05	30 ± 1	23 ± 3	21 ± 1	15 ± 2	1.7 ± 0.4	2.0 ± 0.5

Data is shown as average ± SD of n = 3 independent experiments

7. Determination of uPAR-Binding Affinity (K_D Values)

Purpose: The uPAR-binding affinity of the radiopeptides were determined to investigate the impact of structural changes on the receptor binding.

Methods: HEK-uPAR cells were seeded in poly-D-lysine-coated 48-well plates (0.25×10^6 in 0.5 mL per well) using DMEM cell culture medium with supplements. The cells were incubated at 37 °C and 5% CO_2 overnight to allow adhesion and growth. After removing the supernatant, the well plates with HEK-uPAR cells were placed on ice to prevent internalization of the radiopeptides. The cells were rinsed with cold PBS (0.5 mL, pH 7.4) followed by the addition of DMEM cell culture medium without supplements (450 µL/well) in the presence or absence of AE105 (final concentration 40 µM) as a receptor-blocking agent. The radiopeptides (5 MBq/nmol) were diluted in PBS to obtain peptide concentrations ranging from 10–16000 nM. From each dilution, 50 µL were added to each well to obtain final peptide concentrations of 1 nM to 1600 nM. The cells were incubated at 4 °C for 1 h before rinsing twice with PBS (0.5 mL, pH 7.4). The cells were lysed using an aqueous NaOH solution (1 M, 600 µL) and the lysates were transferred to RIA tubes for counting the activity in a γ-counter (1480 Wizard, PerkinElmer). The K_D values were determined by plotting specific binding (total binding minus unspecific binding) against the molar concentration of the added radiopeptide using GraphPad Prism (version 8.3.1). the results were then expressed as average K_D values ± SD of n = 3 independent experiments.

Results: The results are reported in the main article.

8. Biodistribution Studies

Purpose: Quantitative biodistribution studies were performed in HEK-uPAR xenografted CD1/nude mice after injection of the newly designed radiopeptides to evaluate the impact of the albumin-binding entity on the in vivo distribution of the novel radiopeptides compared to that of [^{177}Lu]Lu-DOTA-AE105.

Methods: The methods are reported in the main article.

Results: The results are reported in the main article and listed in Tables S7–S12.

Table S7. Biodistribution Data and Xenograft-to-Background Ratios Obtained in HEK-uPAR Xenograft-Bearing Mice After Injection of [^{177}Lu]Lu-DOTA-AE105

	**[^{177}Lu]Lu-DOTA-AE105**	
	**4 h p.i.**	**24 h p.i.**
Blood	0.08 ± 0.01	<0.05
Heart	0.06 ± 0.01	<0.05
Lung	0.30 ± 0.02	0.21 ± 0.01
Spleen	0.18 ± 0.02	0.23 ± 0.04
Kidneys	1.5 ± 0.1	1.3 ± 0.1
Stomach	0.15 ± 0.03	0.17 ± 0.03
Pancreas	0.06 ± 0.01	0.06 ± 0.01
Intestines	0.31 ± 0.03	0.18 ± 0.02
Liver	0.10 ± 0.01	0.17 ± 0.01
Muscle	<0.05	<0.05
Bone	0.38 ± 0.03	0.84 ± 0.10
HEK-uPAR xgft	0.87 ± 0.05	0.40 ± 0.19
Salivary glands	0.09 ± 0.01	0.09 ± 0.01
Brain	<0.05	<0.05
Xenograft-to-kidney	0.58 ± 0.03	0.33 ± 0.14
Xenograft-to-blood	11 ± 1	32 ± 13
Xenograft-to-liver	8.6 ± 0.4	2.4 ± 0.9

Decay-corrected data is shown as average ± SD of n = 3 animals of % injected activity per gram (IA/g) tissue.

Table S8. Biodistribution Data and Xenograft-to-Background Ratios Obtained in HEK-uPAR Xenograft-Bearing Mice After Injection of [^{177}Lu]Lu-uPAR-01

	**[^{177}Lu]Lu-uPAR-01**	
	**4 h p.i.**	**24 h p.i.**
Blood	16 ± 1	4.5 ± 0.2
Heart	5.7 ± 0.2	1.9 ± 0.1
Lung	9.4 ± 1.0	3.3 ± 0.3
Spleen	2.7 ± 0.1	1.7 ± 0.2
Kidneys	5.6 ± 0.2	4.2 ± 0.8
Stomach	2.0 ± 0.1	0.78 ± 0.08
Pancreas	2.0 ± 0.3	0.77 ± 0.04
Intestines	2.3 ± 0.3	0.94 ± 0.04
Liver	6.4 ± 0.3	1.8 ± 0.2
Muscle	1.4 ± 0.2	0.57 ± 0.07
Bone	1.9 ± 0.1	0.81 ± 0.01
HEK-uPAR xgft	9.2 ± 0.2	5.8 ± 0.6
Salivary glands	3.6 ± 0.3	1.5 ± 0.1
Brain	0.32 ± 0.02	0.10 ± 0.01
Xenograft-to-Kidney	1.6 ± 0.1	1.4 ± 0.3
Xenograft-to-Blood	0.57 ± 0.01	1.3 ± 0.1
Xenograft-to-Liver	1.4 ± 0.1	3.3 ± 0.3

Decay-corrected data is shown as average ± SD of n = 3 animals of IA/g tissue.

Table S9. Biodistribution Data and Xenograft-to-Background Ratios Obtained in HEK-uPAR Xenograft-Bearing Mice After Injection of [^{177}Lu]Lu-uPAR-02

	[^{177}Lu]Lu-uPAR-02	
	**4 h p.i.**	**24 h p.i.**
Blood	16 ± 1	6.0 ± 0.5
Heart	4.8 ± 0.2	2.5 ± 0.3
Lung	7.2 ± 0.3	4.0 ± 0.2
Spleen	2.2 ± 0.1	2.0 ± 0.2
Kidneys	5.5 ± 0.2	3.6 ± 0.2
Stomach	1.6 ± 0.3	0.69 ± 0.03
Pancreas	1.8 ± 0.1	0.92 ± 0.06
Intestines	1.8 ± 0.2	0.84 ± 0.13
Liver	2.8 ± 0.3	1.6 ± 0.2
Muscle	1.4 ± 0.1	0.90 ± 0.06
Bone	1.8 ± 0.2	1.2 ± 0.1
HEK-uPAR xgft	11 ± 2	10 ± 3
Salivary glands	3.2 ± 0.4	1.8 ± 0.2
Brain	0.34 ± 0.03	0.13 ± 0.02
Xenograft-to-Kidney	2.0 ± 0.2	2.8 ± 0.6
Xenograft-to-Blood	0.71 ± 0.08	1.7 ± 0.4
Xenograft-to-Liver	4.1 ± 0.7	6.2 ± 1.2

Decay-corrected data is shown as average ± SD of n = 3 animals of IA/g tissue.

Table S10. Biodistribution Data and Xenograft-to-Background Ratios Obtained in HEK-uPAR Xenograft-Bearing Mice After Injection of [^{177}Lu]Lu-uPAR-03

	[^{177}Lu]Lu-uPAR-03	
	**4 h p.i.**	**24 h p.i.**
Blood	15 ± 1	13 ± 1
Heart	4.8 ± 0.5	5.2 ± 0.1
Lung	7.5 ± 0.5	7.7 ± 0.7
Spleen	2.2 ± 0.2	3.0 ± 0.2
Kidneys	4.1 ± 0.3	4.7 ± 0.2
Stomach	1.6 ± 0.1	1.8 ± 0.1
Pancreas	1.7 ± 0.1	1.9 ± 0.2
Intestines	1.8 ± 0.1	2.0 ± 0.1
Liver	3.1 ± 0.4	3.2 ± 0.2
Muscle	1.3 ± 0.1	1.5 ± 0.1
Bone	1.5 ± 0.3	1.8 ± 0.3
HEK-uPAR xgft	4.0 ± 0.3	7.5 ± 0.2
Salivary glands	3.4 ± 0.4	3.6 ± 0.2
Brain	0.31 ± 0.05	0.30 ± 0.01
Xenograft-to-Kidney	0.96 ± 0.06	1.6 ± 0.1
Xenograft-to-Blood	0.26 ± 0.02	0.58 ± 0.01
Xenograft-to-Liver	1.3 ± 0.2	2.4 ± 0.2

Decay-corrected data is shown as average ± SD of n = 3 animals of IA/g tissue.

Table S11. Biodistribution Data and Xenograft-to-Background Ratios Obtained in HEK-uPAR Xenograft-Bearing Mice After Injection of [^{177}Lu]Lu-uPAR-04

	[^{177}Lu]Lu-uPAR-04	
	**4 h p.i.**	**24 h p.i.**
Blood	13 ± 1	7.0 ± 0.9
Heart	4.4 ± 0.6	3.0 ± 0.3
Lung	6.2 ± 0.7	4.3 ± 0.5
Spleen	1.9 ± 0.1	2.3 ± 0.3
Kidneys	5.9 ± 0.4	5.9 ± 1.2
Stomach	1.5 ± 0.1	1.0 ± 0.1
Pancreas	1.5 ± 0.1	1.0 ± 0.1
Intestines	1.6 ± 0.1	0.99 ± 0.21
Liver	3.9 ± 0.3	3.1 ± 0.4
Muscle	1.0 ± 0.1	0.73 ± 0.04
Bone	1.2 ± 0.1	0.85 ± 0.04
HEK-uPAR xgft	3.6 ± 0.2	4.3 ± 1.2
Salivary glands	2.8 ± 0.2	2.4 ± 0.4
Brain	0.26 ± 0.02	0.14 ± 0.01
Xenograft-to-Kidney	0.62 ± 0.01	0.73 ± 0.15
Xenograft-to-Blood	0.28 ± 0.01	0.61 ± 0.09
Xenograft-to-Liver	0.93 ± 0.08	1.4 ± 0.2s

Decay-corrected data is shown as average ± SD of n = 3 animals of IA/g tissue.

Table S12. Biodistribution Data and Xenograft-to-Background Ratios Obtained in HEK-uPAR Xenograft-Bearing Mice After Injection of [^{177}Lu]Lu-uPAR-05

	[^{177}Lu]Lu-uPAR-05	
	**4 h p.i.**	**24 h p.i.**
Blood	12 ± 1	4.6 ± 0.3
Heart	4.1 ± 0.2	1.6 ± 0.1
Lung	7.0 ± 0.5	2.6 ± 0.4
Spleen	1.5 ± 0.1	0.82 ± 0.10
Kidneys	3.6 ± 0.2	1.6 ± 0.1
Stomach	1.3 ± 0.1	0.64 ± 0.01
Pancreas	1.4 ± 0.2	0.62 ± 0.07
Intestines	1.6 ± 0.1	0.73 ± 0.07
Liver	5.2 ± 0.6	1.1 ± 0.1
Muscle	1.1 ± 0.1	0.41 ± 0.04
Bone	1.1 ± 0.1	0.67 ± 0.06
HEK-uPAR xgft	5.1 ± 1.0	3.1 ± 0.7
Salivary glands	2.5 ± 0.3	1.1 ± 0.1
Brain	0.27 ± 0.01	0.11 ± 0.02
Xenograft-to-Kidney	1.4 ± 0.2	1.9 ± 0.3
Xenograft-to-Blood	0.42 ± 0.07	0.66 ± 0.12
Xenograft-to-Liver	0.98 ± 0.10	2.9 ± 0.3

Decay-corrected data is shown as average ± SD of n = 3 animals of IA/g tissue.

9. In Vivo Stability Studies

Purpose: The in vivo stability of the uPAR-targeting radiopeptides was investigated in immunocompetent FVB mice by analyzing urine, blood plasma, liver and kidneys.

Methods: FVB mice (n=2) were injected with the radiopeptides (25 MBq, 0.5 nmol, in 100 μL 0.05% bovine serum albumin in saline) and urine was collected at 1 h and 4 h p.i.. After the second collection, the mice were sacrificed and blood samples were taken from the heart. Liver and kidney samples were obtained. Additionally, two mice injected with [^{177}Lu]Lu-DOTA-AE105 were euthanized 1 h p.i. for the same sample collection. The blood sampled from the heart was centrifuged (200 rcf, 4 °C, 10 min) to obtain blood plasma. A drop of urine and blood plasma were analyzed using the TLC method described above. Both kidneys and a part of the liver (~200 mg) were cut into pieces, homogenized using 200–300 μL buffer solution (ice-cold methanol with 0.025% NH_4OH) and centrifuged (2300 rcf, 4° C, 5 min) as previously described by Beyer *et al.*[2] The supernatant of each sample was developed using the TLC system described above. The injection solution of the respective radiopeptide was spotted as a control on each TLC plate. The TLC plates were exposed to a phosphor screen (Super resolution screen PSR10450013, PerkinElmer) followed by development using a storage phosphor system

(Cyclone Plus, PerkinElmer). The quantification of the signals was carried out using the OptiQuant software (version 5.0, Bright Instrument Co Ltd., PerkinElmer). The obtained chromatograms were analyzed by determination of the peak area of the radiolabeled peptides as well as degradation products of unknown structure. The quantity of the intact product was expressed as percentage of the sum of integrated peak areas of the entire chromatogram.

Results: The results are reported in the main manuscript, Figure S11 and Table S13. Additonal data obtained at 1 h after injection of [^{177}Lu]Lu-DOTA-AE105 revealed that no detectable amount of intact radiopeptide remained in the analyzed probes (Figure S11).

Table S13. Metabolic Stability of Radiopeptides after Injection into FVB Mice

		Blood	Liver	Kidneys	Urine	Urine
		4 h p.i.	4 h p.i.	4 h p.i.	1 h p.i.	4 h p.i.
[^{177}Lu]Lu-DOTA-AE105	intact radiopeptide	<1.0%	<1.0%	<1.0%	1.4%	2.3%
	radiometabolite	100%	100%	100%	99%	98%
[^{177}Lu]Lu-uPAR-01	intact radiopeptide	23%	39%	32%	8.5%	6.2%
	radiometabolite	77%	61%	68%	92%	94%
[^{177}Lu]Lu-uPAR-02	intact radiopeptide	69%	53%	50%	8.1%	81%
	radiometabolite	31%	47%	50%	92%	19%
[^{177}Lu]Lu-uPAR-03	intact radiopeptide	99%	67%	91%	<1.0%	7.9%
	radiometabolite	1.2%	33%	9.4%	100%	92%
[^{177}Lu]Lu-uPAR-04	intact radiopeptide	30%	9.9%	12%	<1.0%	2.3%
	radiometabolite	70%	90%	88%	100%	98%
[^{177}Lu]Lu-uPAR-05	intact radiopeptide	18%	54%	20%	7.7%	6.1%
	radiometabolite	82%	46%	80%	92%	94%

Data is shown as average from samples of (n = 2) mice per radiopeptide

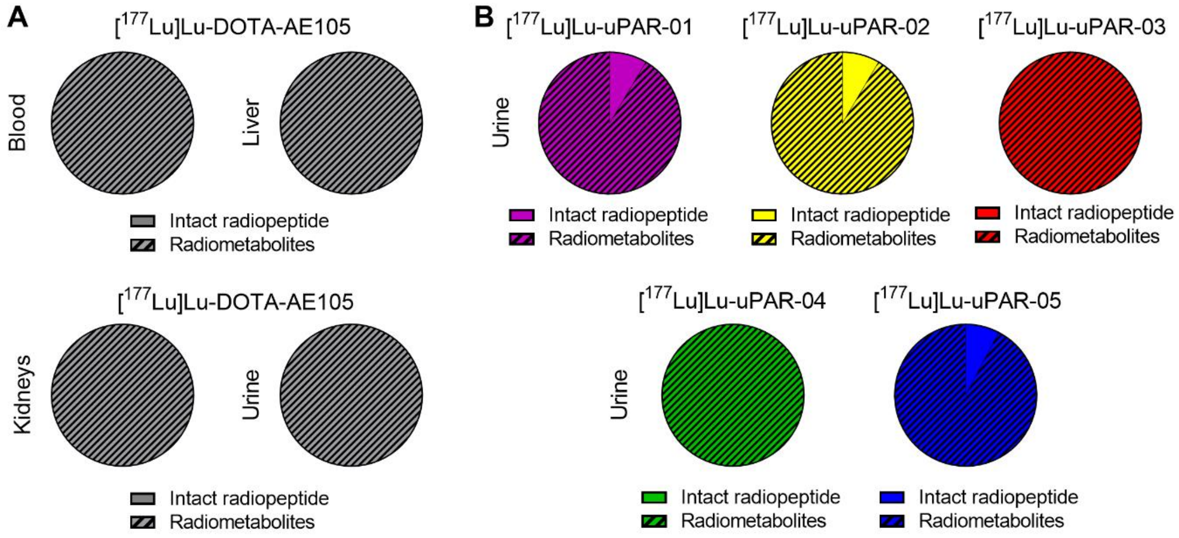

Figure S11. Metabolic stability of radiopeptides 1 h p.i. in FVB mice. (A) Presence of radiometabolites 1 h p.i. of [^{177}Lu]Lu-DOTA-AE105 found in blood, liver, kidneys and urine. (B) Presence of radiometabolites in urine of FVB mice 1 h p.i. of albumin-binding radiopeptides.

References

(1) Persson, M.; Madsen, J.; Ostergaard, S.; Jensen, M. M.; Jorgensen, J. T.; Juhl, K.; Lehmann, C.; Ploug, M.; Kjaer, A. Quantitative PET of human urokinase-type plasminogen activator receptor with ^{64}Cu-DOTA-AE105: implications for visualizing cancer invasion. *J Nucl Med* **2012**, *53* (1), 138-145. DOI: 10.2967/jnumed.110.083386

(2) Beyer, D.; Vaccarin, C.; Deupi, X.; Mapanao, A. K.; Cohrs, S.; Sozzi-Guo, F.; Grundler, P. V.; van der Meulen, N. P.; Wang, J.; Tanriver, M.; et al. A tool for nuclear imaging of the SARS-CoV-2 entry receptor: molecular model and preclinical development of ACE2-selective radiopeptides. *EJNMMI Res* **2023**, *13* (1), 32. DOI: 10.1186/s13550-023-00979-2
